# Enhancement and Distortion in the Temporal Representation of Sounds in the Ventral Cochlear Nucleus of Chinchillas and Cats

**DOI:** 10.1371/journal.pone.0044286

**Published:** 2012-09-18

**Authors:** Alberto Recio-Spinoso

**Affiliations:** Laboratory for Auditory Neuroengineering, IDINE, Universidad de Castilla-La Mancha, Albacete, Spain; University of Salamanca - Institute for Neuroscience of Castille and Leon and Medical School, Spain

## Abstract

A subset of neurons in the cochlear nucleus (CN) of the auditory brainstem has the ability to enhance the auditory nerve's temporal representation of stimulating sounds. These neurons reside in the ventral region of the CN (VCN) and are usually known as highly synchronized, or high-sync, neurons. Most published reports about the existence and properties of high-sync neurons are based on recordings performed on a VCN output tract—not the VCN itself—of cats. In other species, comprehensive studies detailing the properties of high-sync neurons, or even acknowledging their existence, are missing.

Examination of the responses of a population of VCN neurons in chinchillas revealed that a subset of those neurons have temporal properties similar to high-sync neurons in the cat. Phase locking and entrainment—the ability of a neuron to fire action potentials at a certain stimulus phase and at almost every stimulus period, respectively—have similar maximum values in cats and chinchillas. Ranges of characteristic frequencies for high-sync neurons in chinchillas and cats extend up to 600 and 1000 Hz, respectively. Enhancement of temporal processing relative to auditory nerve fibers (ANFs), which has been shown previously in cats using tonal and white-noise stimuli, is also demonstrated here in the responses of VCN neurons to synthetic and spoken vowel sounds.

Along with the large amount of phase locking displayed by some VCN neurons there occurs a deterioration in the spectral representation of the stimuli (tones or vowels). High-sync neurons exhibit a greater distortion in their responses to tones or vowels than do other types of VCN neurons and auditory nerve fibers.

Standard deviations of first-spike latency measured in responses of high-sync neurons are lower than similar values measured in ANFs' responses. This might indicate a role of high-sync neurons in other tasks beyond sound localization.

## Introduction

In response to low-frequency sinusoidal sounds, auditory nerve fibers (ANFs) generate trains of action potentials, or spikes, that are synchronized to the stimulating waveform—a property usually known as phase locking [1], [2]. Temporal processing is a key function of the auditory system, as it is relevant for the detection of features important for speech comprehension [3].

Quantification of phase locking is usually done using the vector strength (*r*) metric [4], where 0≤*r*≤1. (A value of *r* = 1 indicates perfect synchronization to one phase of the stimulus waveform; *r* = 0 implies a lack of preference to any particular stimulus phase.) In cat, maximum *r* values, *r*_max_, hold relatively constant for stimulus frequencies below 1 kHz and decrease gradually up to around 5 kHz [5], [6]. Responses to characteristic frequency (CF: frequency of lowest threshold) tones by cat ANFs yield *r*_max_≤0.9 [5], [6], [7], [8]. In chinchilla, ANF responses to CF tones also produce *r*_max_≤0.9 [9], [10].

Phase locking is also a property of neurons in the cochlear nucleus (CN)—the brainstem structure to which ANFs project. Different CN regions exhibit different amounts of phase locking [11], [12], with neurons in the ventral portion of the CN (VCN) being more adept at temporal processing than those in the dorsal CN. A discrepancy exists, however, among published studies regarding synchronization limits of VCN neurons with CFs below approximately 1 kHz. Some studies have found that phase-locking strength exhibited by VCN neurons deteriorates or remains constant relative to ANFs [11], [13]–[16]. The work of others [6], [7], [17], [18], however, has shown an enhancement in phase locking relative to the auditory nerve (AN), at least for CFs below 1 kHz.

AVCN neurons in cat that show an improvement in phase locking over auditory nerve fibers often exhibit *r*_max_>0.9 in their responses to CF tones—greater than *r*_max_ values computed from ANF responses to CF tones. These neurons, which are usually referred to as highly synchronized (or high-sync), are also capable of firing action potentials at almost every stimulus cycle, a property called entrainment [6], [19], [20]. Variability in interspike intervals, such as in ANF's spike trains, yields lower entrainment levels while increasing the quality of the stimulus waveform representation [20], [21].

Temporal processing enhancement in the VCN seems less prominent in rodents than in cats [19]. Moreover, published works in guinea pigs [16] and rats [18] show a lack of high-sync VCN neurons in their recordings (i.e., *r*_max_≤0.9). It is shown here that the strength of phase-locking in the chinchilla's VCN resembles the cat's, at least for CFs<600 Hz, thus enhancing the synchronization capabilities of ANFs. In other words, there exist highly synchronized neurons in chinchilla and some of those neurons also exhibit significant entrainment to the stimulus. The improvement in synchronization, however, comes with greater distortion in the representation of the stimulating waveform.

## Methods

### Surgical procedures

Chinchillas (average weight = 500 grams) and cats (average weight = 3 Kg) were initially anesthetized with intraperitoneal injections of sodium pentobarbital (75 mg/Kg, i.p., and 50 mg/Kg, i.p., for chinchillas and cats, respectively). A level of surgical anesthesia was maintained by additional i.p. injections of pentobarbital in chinchillas and via a catheter inserted in the femoral vein in cats. The rest of the surgical procedure was very similar for both species. A thermostatically controlled heating blanket maintained body temperature at 37°C. After insertion of a tracheal cannula, the left ear was removed and the bulla was vented with 20 cm of a 1-mm plastic tube. Following removal of the overlying cerebellum, the cochlear nucleus was covered with agar. To further reduce brain pulsations, a chamber was mounted over the skull opening and filled with mineral oil. Two scales were positioned along the cat CN, one scale in chinchilla, to record the position of the electrode. Auditory nerve recordings were performed in some experiments (mostly cats). For this purpose, the auditory nerve was approached following retraction of the cochlear nucleus by inserting cotton balls between the CN and the temporal bone.

Several recordings in chinchillas were obtained from axons in the ventral acoustic stria (VAS), which is one of the three output tracts of the CN. The VAS was accessed dorsally by inserting a microelectrode at least 4 mm through the floor of the fourth ventricle [22].

Some of the data were collected from cats that were part of labeling experiments published elsewhere [23], [24]. Based on histological reconstructions in some of the animals, it was judged that the recordings originated from the nerve root area and the caudal end of the AVCN.

### Experimental protocol

A RadioShack SuperTweeter speaker was used to present the acoustic stimuli in a closed ear coupler. A calibrated probe tube was inserted very close to the eardrum. The other end of the probe tube was connected to a ½″ Bruel and Kjær condenser microphone and was amplified 60 dB. Acoustic stimuli were calibrated from 50–24000 Hz in 50-Hz steps in the chinchilla and from 50–40000 Hz in 50-Hz steps in the cat.

KCl-filled micropipettes, with impedances of 10–40 MΩ, were used to record single-unit activity from either CN units or ANFs. Spike times relative to stimulus onset were determined using a level detector (i.e., the output of an oscilloscope trigger) and stored digitally at a 1-µs resolution. A high-intensity FM sweep was used as search stimulus for neurons. Responses to this search stimulus yielded an initial estimate of the CF of the neuron. The neuron's receptive field (i.e., a family of iso-intensity curves) was determined using five or ten 50-ms tone pips with intensities varying between 0 and 90 dB SPL, in 10 dB SPL increments. Frequency separation was usually 100 Hz for CFs<2000 Hz or 200 Hz for CFs>2000 Hz. Threshold and final CF estimates were determined by analysis of the receptive field. Post-stimulus time histograms (PSTHs) at CF were obtained using 250 repetitions of a 50 ms tone pip, usually at 60 dB SPL. For most neurons in this study, this level corresponds to 30 to 50 dB relative to threshold. If necessary, a PSTH at CF was also obtained using an 80-dB SPL tone. Rate-vs.-level curves at CF were also obtained from 0 to 90 dB SPL in 5-dB SPL steps.

Analysis of the responses of chinchilla VCN neurons (CF<1000 Hz) to synthetic vowel stimuli are presented here. These data have not been published before but were collected as part of a previous study [22]. Vowels were generated using the cascade branch of a Klatt synthesizer (1980). The fundamental frequency, *f_0_*, was 100 Hz for most neurons except in one case (*f_0_* = 181.6 Hz). Only responses to vowels /*i*/ (as in *heed*) and /*ε*/ (as in *bet*) will be discussed here. First (F_1_) and second (F_2_) formant frequencies for /*i*/ are 300 and 2300 Hz, respectively. F_1_ and F_2_ frequencies for /*ε*/ are 500 and 1800 Hz, respectively. Each vowel stimulus (duration = 150 ms) was presented 200 times, with a repetition period of 350 or 500 ms at three intensity levels: 20, 40 and 60 effective dB SPL. (The measure of sound intensity, effective dB SPL, is computed by summing energy contributions from each frequency band in the speech waveform. Each energy contribution is a proportion of the maximum dB SPL taken from the acoustic tone calibration.)

Whispered vowels (/*i*/, /*u*/ and /*a*/) were recorded in a soundproof room from two native speakers of American English (male and female) and presented to anesthetized cats. The microphone used for the recordings was a ½″ Bruel and Kjær condenser microphone and the sampling rate was 20 kHz. Each vowel (duration = 300 ms) was presented 100 times at a rate of 1/sec at 40 and 60 dB effective SPL at a playback rate of 50 kHz (following resampling). Only responses to the female /*i*/ sound will be presented in this report.

### Temporal analysis and unit classification

Because glass micropipettes were used to record neural activity, the segregation of primary and secondary neurons posed a problem. (This was not a concern for VAS recordings.) Shapes of PSTHs as well as certain response statistics of CN neurons are well known [11], [13], but that classification is based on responses to moderate- to high-frequency stimulation (>1 kHz). A neuron was classified as originating from the VCN if it satisfied at least one of the three criteria described here:

1. Responses to CF tones yielded *r*>0.9. The 0.9 value was used as a criterion based on published *r*_max_ for ANF responses in cats [5], [6] and chinchillas [9], [10].The statistical significance (*p*<0.001) of *r* was evaluated using the Rayleigh test [25].
2. The shape of the action potential was bipolar [26].
3. There was a rate reduction below the spontaneous rate activity in response to single off-CF tones with increasing intensity [24].

Responses of VCN neurons with CFs in the 1–2 kHz range are also reported here. In response to CF tones, these neurons have PSTHs with one of the two following shapes: primarylike-with-notch (PLN) or an onset with an L-shaped PSTH (OnL). Based on labeling experiments [6], [11], [24], those PSTHs are primarily associated with globular bushy cells.

Units deemed VCN neurons using the criteria expressed above were classified into three mutually exclusive groups:

Group 1: Neurons with *r*>0.9 in their responses to CF tones.
  (Group 1 corresponds to highly synchronized neurons.)
Group 2: Neurons with r≤0.9 in response to CF tones but able to synchronize strongly (i.e., *r*>0.9) to certain off-CF tones.
Group 3: Neurons with r≤0.9 in response to single tones, regardless of the stimulus frequency.

Regularity analysis [13] was performed on interspike intervals (ISIs) computed from ANFs' and VCN neurons' responses to CF tones. The coefficient of variation (*CV*) of the ISI was used as an indicator of the regularity of the response [13]. *CV* is defined as the ratio of the standard deviation of the ISI to its mean value. In cats, average *CV*s obtained from responses of ANFs and high-sync neurons are 0.68 and 0.45, respectively. Statistical analyses show significant differences between the *CV*s of ANFs and high-sync neurons (Kolmogorov-Smirnoff test, *P*>0.001). In chinchillas, the combined sample of Groups 1 and 2 neurons also exhibit *CV*s (μ = 0.45) that are smaller than *CV*s (μ = 0.65) computed from ANF responses (Such differences are statistically significant as determined by the Kolmogorov-Smirnoff test, *P*>0.001). *CV*s from the population of chinchilla Group 3 neurons are also smaller (μ = 0.38) than *CV* values in ANFs (Kolmogorov-Smirnoff test, *P*>0.001). CFs of all the units used in the above analyses were below 2 kHz. The fact that units classified as VCN neurons have lower *CV*s than ANFs validates the aforementioned criteria to segregate primary from second-order neurons.

Entrainment indices were computed from ISI histograms. The index is defined as the ratio of the number of intervals that fall in a window located at 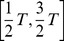,where *T* = 1/CF, to the total number of intervals [6]. For responses perfectly entrained to the stimulus, this index equals 1.

The quality of the representation of the stimulus waveform in the discharges of ANFs and VCN neurons was evaluated using a measure of distortion. Distortion in the neural response to a CF tone was measured by computing the power spectrum density (PSD) of the spike train. Total harmonic distortion (THD) was defined as the sum of the powers of the first eight harmonics to the power of the spectral component at CF:
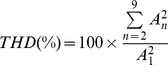
(1)where 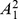 and 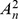 represent the power of the spectral component at CF and its harmonics, respectively. For responses to single tones, *A*_1_ corresponds to the amplitude at the tone frequency. Similarly for responses to vowel stimuli, *A*_1_ corresponds to the amplitude at the F_1_ frequency.

Temporal analysis of neural responses to vowel sounds was performed using period histograms and shuffled autocorrelograms (SACs). The duration of the cycle used to construct period histograms was set to 1/*f*_0_. SACs were constructed using MATLAB routines provided in [27]. The analysis windows for the construction of the SACs were 50–150 ms and 50–300 ms for synthetic and whispered spoken vowels, respectively. The duration of the binwidth was 50 µs for all the SACs. Correlation indices (CIs), which correspond to the peak value of the normalized SACs [7], were computed to quantify the temporal structure in the neural responses to vowel sounds.

### Ethics statement

Experiments were performed at the Department of Physiology, University of Wisconsin – Madison, where the author was a research scientist. All procedures were approved by the Animal Care and Use Committee of the University of Wisconsin-Madison (Protocol ID: M00205).

## Results

Responses were obtained from VCN neurons and ANFs in 29 chinchillas and 9 cats. Because of the small number of chinchilla ANFs collected for this research, *r* values from a published study (Fig. 7 in [9]) were used for comparison purposes.

### Responses to single tones of highly synchronized neurons in chinchilla and cat

**Figure 1:**
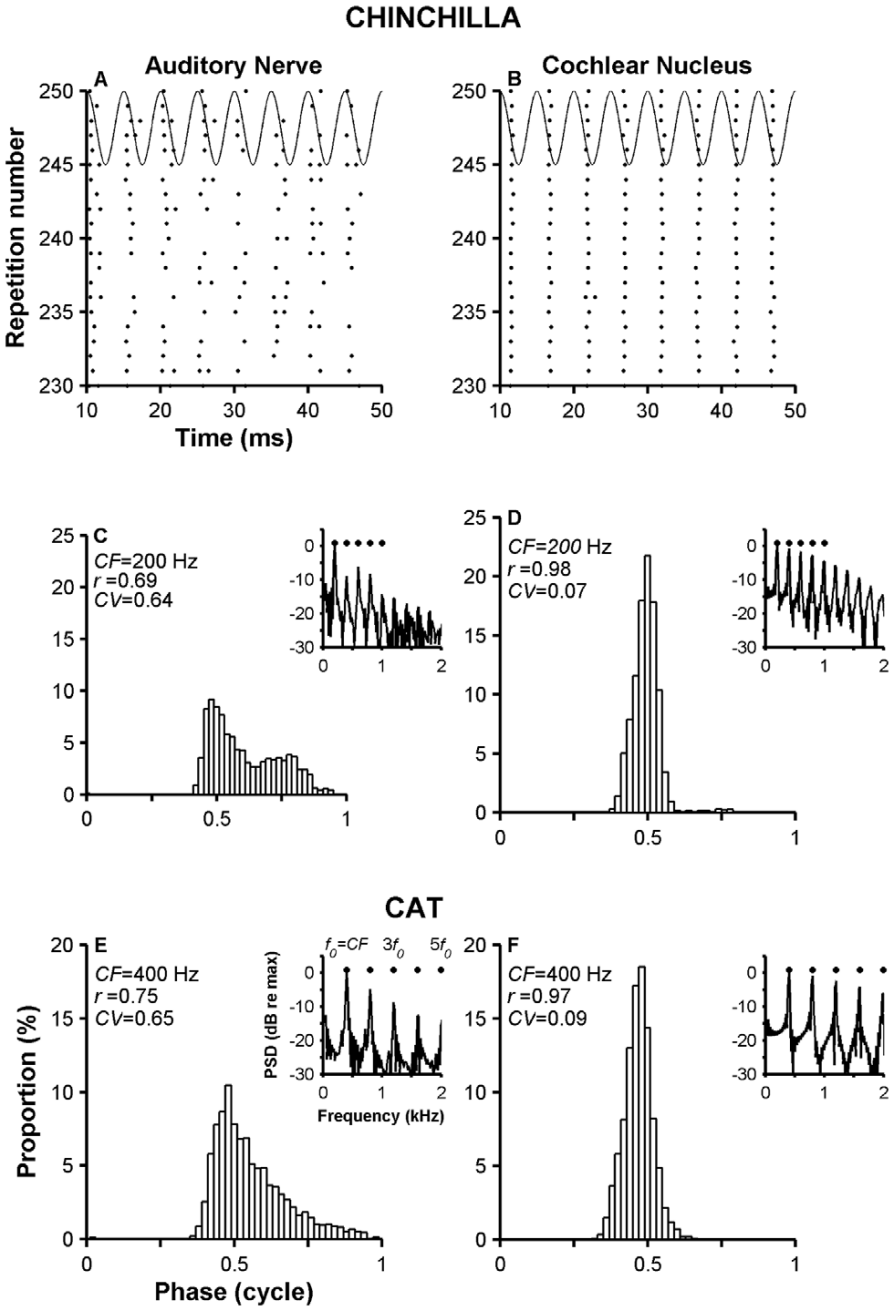
Enhancement of synchrony and rate relative to auditory nerve in chinchilla and cat. Panels A and B display dot raster plots for the responses of an auditory nerve fiber and a cochlear nucleus neuron, respectively, in chinchilla. Only the neural responses obtained in the last 20 stimulus presentations are shown. Stimulus waveforms with an arbitrary phase are superimposed on panels A and B (continuous traces). Panels C and D contain period histograms obtained from responses to CF tones of the ANF and the VCN neuron, respectively. Panels E and F display period histograms obtained from responses of one ANF and one VCN neuron, respectively, in cat. Power spectrum densities (PSDs) of the responses shown in A and B are displayed in the insets C and D, respectively. The first dot in every inset is placed at the frequency corresponding to CF ( = *f*_0_). Period histograms were shifted to make their maximum value occur at 0.5.

Figures 1A and 1B show dot raster plots of the responses of an ANF and a VCN neuron, respectively, to a CF tone. Whereas the chinchilla VCN neuron is well locked to one stimulus phase (continuous single trace in Fig. 1B), responses of the ANF in the same species are less synchronized to the stimulus waveform (continuous trace in Fig. 1A). Vector strength values computed from the ANF's and VCN neuron's responses to the 200-Hz tone were 0.69 and 0.98, respectively. Because of its vector strength value, the VCN neuron was classified as “highly synchronized.”

Discharge patterns of ANFs also show a larger amount of randomness in the interspike intervals than high-sync neurons. A consequence of this jitter can be seen in the broad shape of the period histogram of the responses (Fig. 1C) and in the *CV* value ( = 0.64). By contrast, the period histogram of the high-sync neuron is narrow (Fig. 1D) and has a small *CV* value ( = 0.07). Similar differences occur between period histograms of responses of an ANF and a VCN neuron in cat (Figs. 1E and 1F, respectively).

The ability of a high-sync neuron to fire approximately one action potential per stimulus phase, and with little variability in the spike's time of occurrence, is also evident in Fig. 1B. In fact, near perfect entrainment ( = 0.99) to the stimulus waveform was measured from the response of the high-sync neuron. The entrainment index for the ANF's response was lower ( = 0.57).

**Figure 2:**
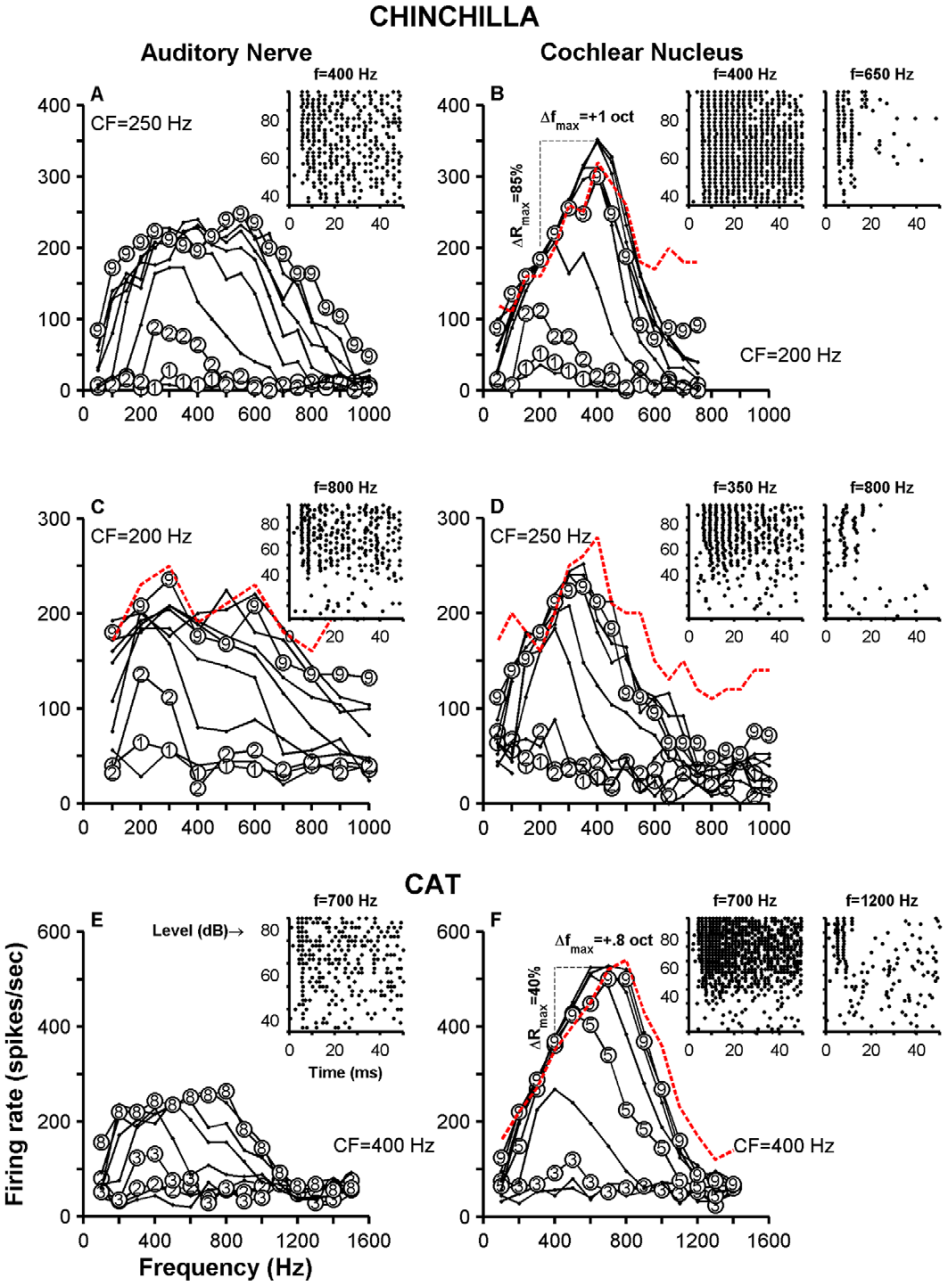
Receptive field maps of ANFs and VCN neurons. Top two rows display iso-intensity curves collected in chinchillas. Panels E and F show iso-intensity curves measured in cats. Data from ANFs are in panels A, C and E. VCN units data are in panels B, D and F. Stimulus levels are indicated in some of the curves (e.g., 1 = 10 dB SPL; 4 = 40 dB SPL). Insets in every panel show dot raster plots at several stimulus levels (5 repetitions per level) indicated in the ordinates. Stimulus frequencies are indicated above each dot raster plot. Firing rates, except for those indicated by the red dashed lines, were measured during the stimulus duration (0–50 ms). Red dashed lines represent firing rate evoked by 90-dB tones during the first 20 ms of the response.

Insets in Figs. 1C–1F show the results of the PSD analysis of the neural responses. The results consist, approximately, of a series of local peaks located at the stimulus frequency (*f_0_* = CF = 200 Hz in Figs. 1C and 1D) and at their harmonics (e.g., 2*f_0_, 3*f_0_, etc.), as indicated by the dots in the insets. (For the insets in Figs. 1E and 1F, *f_0_* = CF = 400 Hz.) The spectral representation at *f_0_* is more predominant for ANFs than for VCN neurons. For example, the amplitudes at *f_0_* and 2*f_0_ are closer in value for VCN neurons (insets in Figs. 1D and 1F) than for ANFs (insets in Figs. 1C and 1E). In Fig. 2, THDs were higher for the two VCN neurons (291% and 228% in chinchilla and cat, respectively) than for the two ANFs (62% and 52% in chinchilla and cat, respectively).

Also notable are the differences in receptive field plots computed from the responses of ANFs and VCN neurons. Although all the receptive field plots in Fig. 2 are asymmetric around CF, such asymmetries are magnified in VCN neuron responses in part because of the large firing rates evoked by some above-CF tones, which can be much larger than rates produced by CF tones. Such differences in firing rates (e.g., ΔR_max_ = 85 and 40% in Figs. 2B and 2F, respectively) are responsible for shifts in the “center of gravity” of the receptive field (Δf_max_ = 1 and 0.8 octaves in Figs. 2B and 2F). Asymmetric receptive fields are also a property of ANFs (Figs. 2A, 2B and 2C) but less noticeable. The large firing rates measured from responses of VCN neurons in Fig. 2 probably arise from the tendency of those neurons to entrain to the stimulus waveform, even for frequencies different from CF [24]. Dot raster plots at several intensity levels displayed in the insets in Figs. 2A and 2B reveal the greater ability of the VCN neuron to follow the stimulus period compared to the ANF, which translates to a higher firing rate for the VCN neuron, especially for stimulus levels above 40 dB SPL. The same observation applies to the results in the cat (insets in Figs. 2E and 2F).

In response to certain above-CF tones, VCN neurons in Fig. 2 fire more vigorously near the response onset (i.e., before 20 ms) than during the steady-state part. This behavior is clearly seen in the dot raster plot of the responses to 800-Hz tones of the neuron in the Fig. 2D inset. For stimulus levels above 40–50 dB SPL, action potentials are fired only near the onset. This period of activity is followed by a “quiet” period, during which even the spontaneous activity is inhibited. Dot raster plots in Figs. 2B and 2D in responses to 650-Hz and 1200-Hz tones, respectively, display a similar pattern. Red dashed lines in Fig. 2 indicate firing rates computed during the initial 20 ms of the response to 90-dB tones of different frequencies. For VCN units, firing rates computed during the response onset (0–20 ms) resemble those measured during the complete stimulus duration (0–50 ms) for stimulus frequencies below CF. For stimulus frequencies above CF, onset responses are larger than responses measured during the stimulus duration.

**Figure 3:**
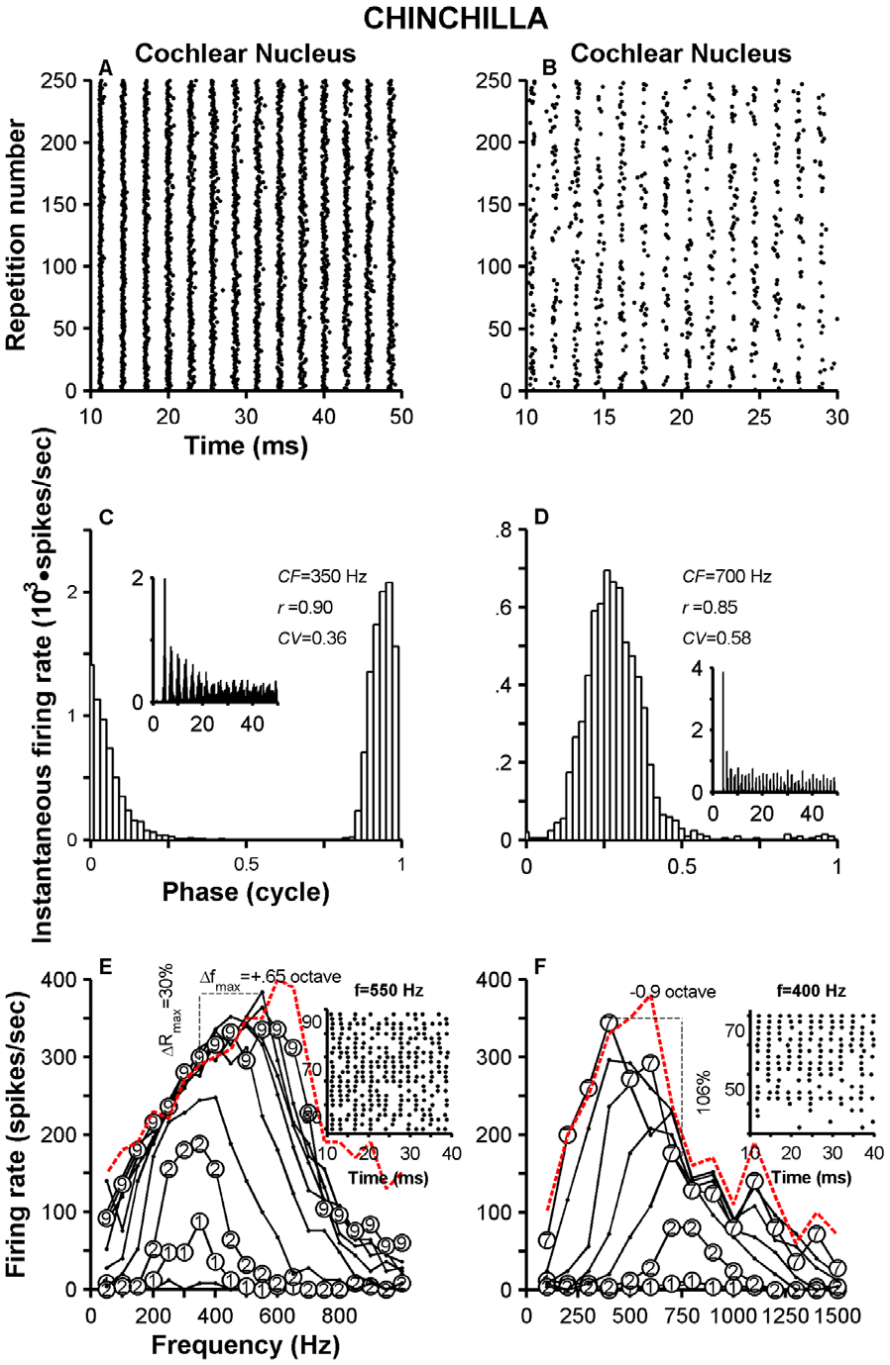
Single-tone responses of two chinchilla neurons. Panels A, C and E display, respectively, a dot raster plot, a period histogram and a family of iso-intensity curves computed from the responses of a VCN neuron (CF = 350 Hz, Group 1). Similar results for another VCN neuron (CF = 700 Hz, Group 2) are in panels B, D and F. Insets in C and D show PSTHs from responses to tones whose frequency is indicated in the panels. The binwidth of each PSTH is 0.25 ms. Insets in E and F illustrate dot raster plots of the responses of the neuron to 500 and 400 Hz tone stimuli, respectively, at levels indicated in the ordinates (5 presentations per level; each ordinate tick indicates an increment of 10 dB). Stimulus level in E and F is indicated for only certain iso-intensity curves. ΔR in E and F indicate the increment in maximum firing rate of the neuron relative to the maximum firing rate at CF. Δf represents the frequency shift (in octaves) of the CF frequency toward the frequency of the maximum firing rate. Firing rates, except for those indicated by the red dashed lines, were measured during the stimulus duration (0–50 ms). Red dashed lines represent onset firing rate evoked by 90-dB tones.

Figures 3A and 3B display dot raster plots of responses of two VCN neurons with different CFs. The variability, or jitter, in firing times of action potentials is more noticeable in the neuron with higher CF (700 Hz, Fig. 3B) than in the neuron with CF = 350 Hz (Fig. 3A). This variability is also evident in the *r* and *CV* values measured from their respective responses (Figs. 3C and 3D). Entrainment to the stimulating waveform is higher in the low-CF neuron (0.9) than in the high-CF one (0.03).

**Figure 4:**
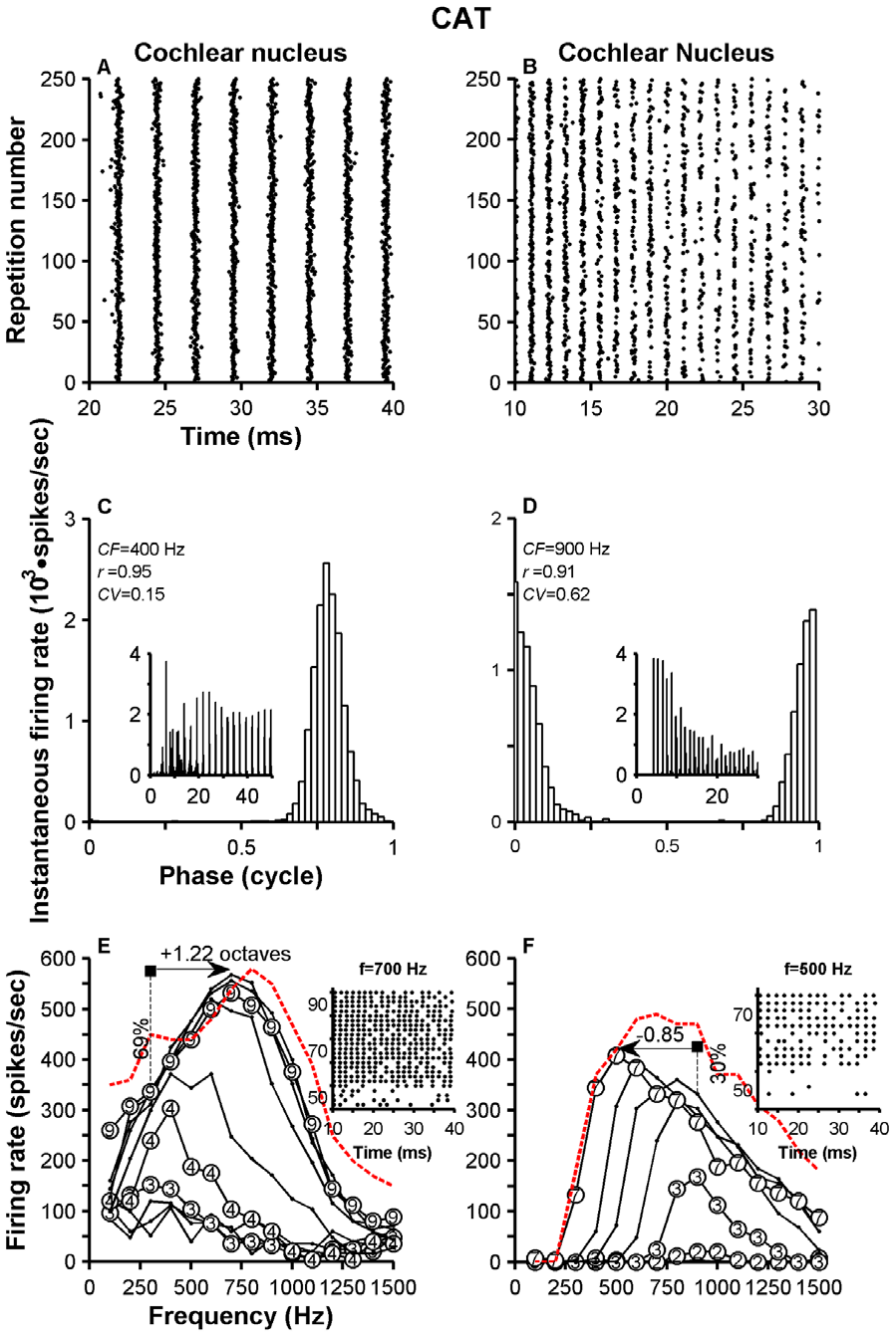
Single tone responses of two cat high-sync neurons. Data from two cat VCN neurons shown in a similar fashion to Fig. 2. Stimulus frequency used to evoke data in panels A and B are 400 and 900 Hz, respectively. Firing rates, except for those indicated by the red dashed lines, were measured during the stimulus duration (0–50 ms). Red dashed lines represent onset firing rates evoked by 90-dB tones, except in panel F (70 dB SPL).

Receptive field plots are shown in Figs. 3E and 3F. In both neurons, maximum firing rates are being evoked by off-CF frequencies. In the case of the neuron whose CF = 350 Hz, the frequency that elicited the maximum rate was 550 Hz, i.e., 0.65 octave *above* CF (Fig. 3E). In the case of the neuron with the highest CF, the maximum firing rate was produced by a 400 Hz tone (Fig. 3F), i.e., almost one octave *below* CF. Such shifts (Δf_max_) are expressed in octaves and can be positive (e.g., Fig. 3E) or negative (e.g., Fig. 3F). Maximum firing rates computed from receptive fields in Figs. 3E and 3F are much larger than maximum rates evoked by CF tones. Those changes, ΔR_max_, are expressed as a percentage relative to maximum CF rate. Qualitatively similar changes in Δf_max_ and ΔR_max_ have been shown in similar plots obtained from responses of chinchilla ANFs (Figs. 2,4 and 6 in [9]). The extent of those changes, however, is much larger in high-sync neurons than in ANFs.

Insets in Figs. 3E and 3F display dot raster plots of the responses of the neurons to 550 and 400 Hz tones, respectively, at several levels (5 repetitions per stimulus level). Each neuron has a tendency to fire around 4 spikes in a 10-ms interval (especially in the inset in Fig. 3E). This entrainment is responsible for the large firing rates at 550 Hz, which produce large asymmetries in the receptive field map.

Figure 4 illustrates the responses of two high-sync neurons recorded in the cat VCN. Response characteristics are similar to those in Fig. 3, with the exception of the higher values for *r* obtained in cat VCN neurons (compare Fig. 3D to Fig. 4D). Entrainment indices obtained from responses in Figs. 4A and 4B were 0.98 and 0.148, respectively.

PSTHs in some insets in Figs. 3 and 4 were obtained from responses to CF tones and resemble histograms of “locker” neurons [6], [11]. With the exception of the PSTH in Fig. 3A, all other histograms display instantaneous firing rates of up to 4000 spikes/sec, near the stimulus onset. Such rates indicate the ability of the respective neurons to fire one spike at the onset of each stimulus presentation with little variability in time of occurrence. (PSTHs were constructed using a binwidth = 0.25 ms.)

Figure 4E also shows a decrease in the firing rate of the neuron below the spontaneous rate for a range of stimulus frequencies above CF (1000–1500 Hz). Single-tone inhibition has been previously found by Rhode [24] in VCN neuron responses and is not a property of ANFs. Dot raster plots in the insets in Figs. 4E and 4F display responses to tones whose frequencies are above CF (700 Hz) and below CF (500 Hz), respectively.

Red dashed lines in Figs. 3 and 4 represent onset (i.e., 0–20 ms) firing rates evoked by 90 dB SPL tones (70 dB SPL in Fig. 4F). Again, onset firing rates evoked by above-CF tones are usually larger than those computed during a 0–50 ms window, especially in cat VCN units. Results from Figs. 2–4 suggest that asymmetries in the receptive fields of certain VCN neurons arise from entrainment and from inhibitory properties of those neurons.

**Figure 5:**
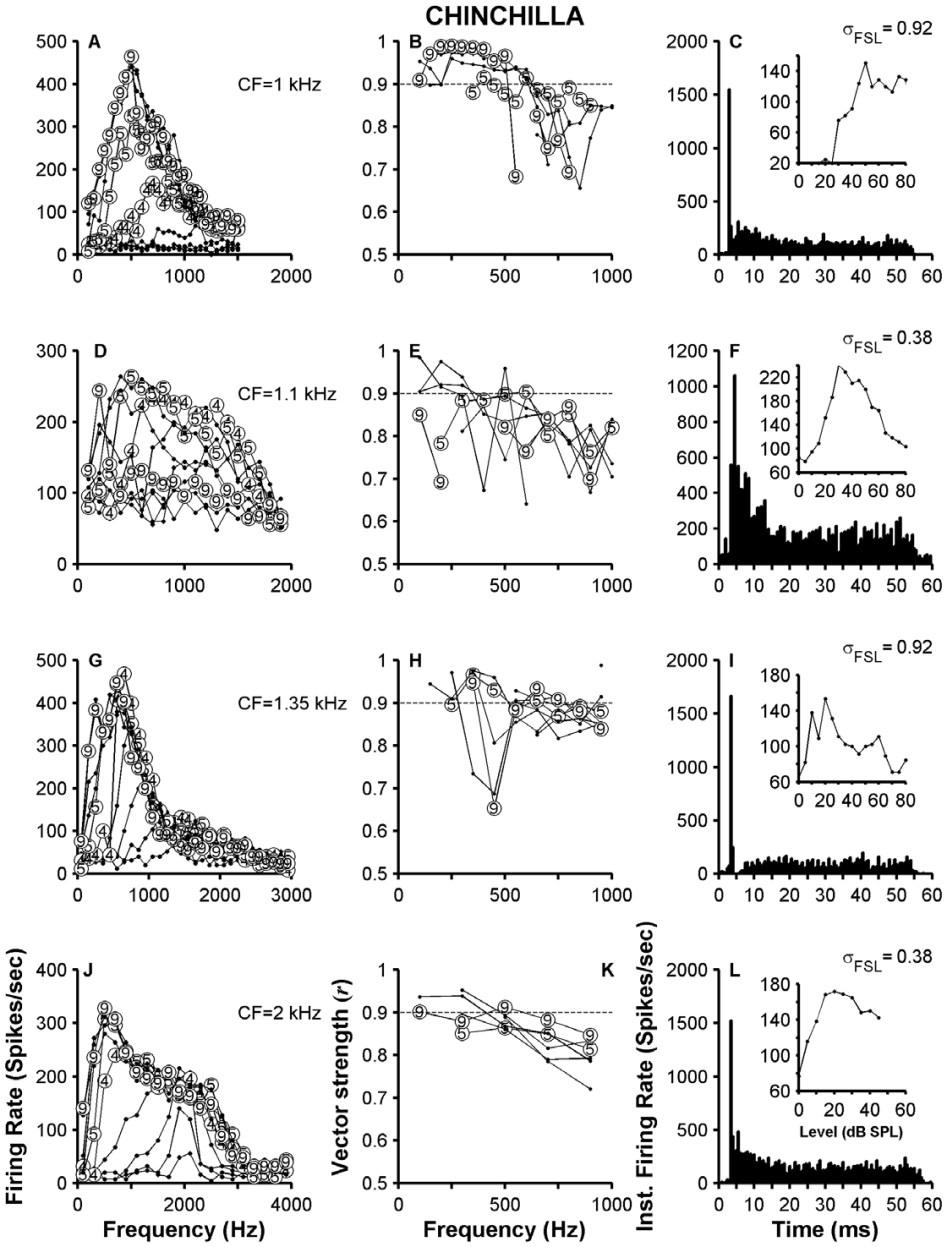
Response properties of PLN/OL neurons in chinchilla. Data across rows come from the same neuron. Left column (i.e., panels A, D, G and J) shows response area curves for four VCN neurons. Center column exhibits response area curves using vector strength values as a response metric. Right column displays PSTHs obtained from responses to CF tones. Standard deviations of first-spike latencies (σ_FSL_) are next to each PSTH.

Figure 5 displays response area curves and PSTHs computed from responses to tones in four chinchilla high-CF VCN neurons (one neuron per row). Results in the second row (panels D, E and F) originate from axonal recordings in the VAS. The shapes of the rate profiles (left column) are asymmetric—a consequence of the elevated firing rates evoked by low-frequency tones. Plots of vector strength as a function of stimulus frequency are in the center column. *r* values evoked by many below-CF tones are above 0.9, which are consistent with previous results [28]. PSTHs were classified as either PL (Fig. 5F), PLN (Fig. 5I) or OnL (Figs. 5C and 5L). Standard deviations of first-spike latencies, σ_FSL_, computed from responses to CF tones are in the rightmost column and are all <1 ms. Firing rates-vs.-level curves (also known as rate-intensity functions, RIFs) obtained from responses to CF tones are shown in the insets in Fig. 5. Except for the RIF in the inset in Fig. 5C, all the RIFs exhibit various degree of nonmonotonic behavior. Approximately 30% of chinchilla VCN neurons in this study were classified as nonmonotonic, using the criterion defined in [16]. None of the cat high-sync neurons in this study were classified as nonmonotonic.

### Response properties of highly synchronized neurons: Population study

Receptive fields of high-sync neurons display changes in their shape as the stimulus level increases (Figs. 2–5). Maximum firing rates evoked by tones whose frequencies are either above or below CF can be larger than the rates evoked by CF tones. This introduces an asymmetry, or change in the “center of gravity,” in the response area that was previously characterized using the metrics Δf_max_ and ΔR_max_ (Figs. 2 and 3).

**Figure 6:**
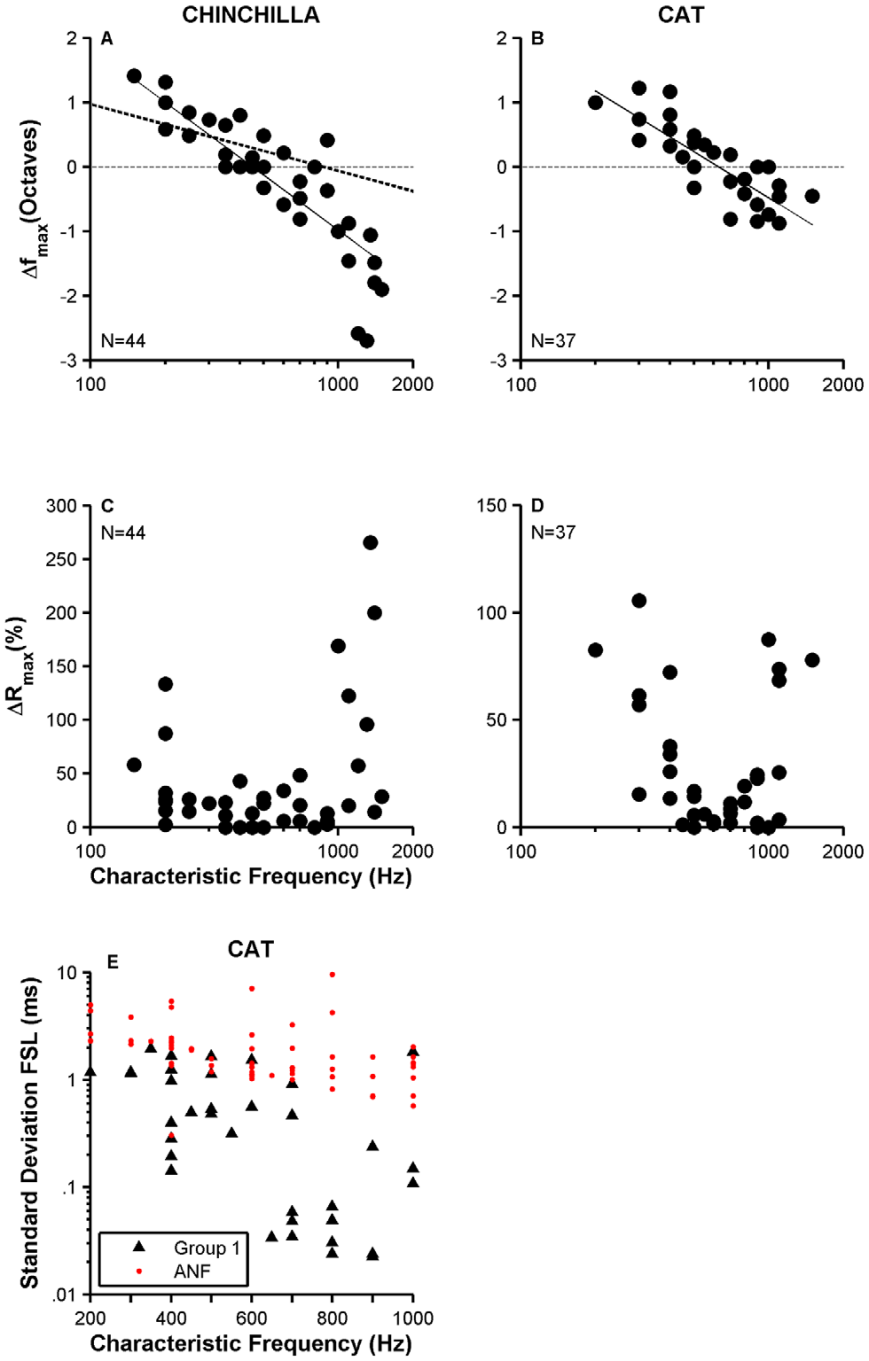
Analysis of receptive field properties and first-spike latencies. Panels A and B illustrate scatter plots of Δf_max_-vs.-CF functions for chinchilla and cat VCN neurons, respectively. Straight continuous lines display a linear fit to the data. Thick dashed line in panel A represents a fit to similar chinchilla ANF data [9]. ΔR_max_-vs.-CF curves for chinchilla and cat neurons are displayed in C and D, respectively. Number of neurons (N) is indicated in each panel. A scatter plot of σ_FSL_ as a function of CF for ANFs (red dots) and high-sync neurons (filled triangles) in cat is in panel E.

Figure 6A displays Δf_max_ values as a function of CF for the sample of chinchilla VCN neurons collected for this report. Δf_max_ is positive for neurons with CF<≈500 Hz and becomes negative for CF>500 Hz. The change in value for Δf_max_ as a function of CF is gradual and near linear (in a semi-logarithmic scale), as indicated by the line fitted to the data (continuous line in Fig. 6A). The bold dashed line in Fig. 6A corresponds to the fit performed on similar ANF data [9]. Larger amounts of frequency shifts of firing rate-vs.-frequency curves are observed in VCN neurons. The flip in polarity in those fits occurs at higher CFs for ANFs (≈900 Hz). Δf_max_-vs.-CF plots in Fig. 6B display a similar behavior for cat VCN neurons, except that Δf_max_ appears less negative than the data shown in Fig. 6A, especially for frequencies above 700 Hz. That means the shift in the center of gravity of the rate-vs.-frequency curves is smaller in cats than in chinchillas, at least for such CFs. Changes in maximum firing rate relative to maximum rate at CF, ΔR_max_, for VCN neurons in chinchilla and cat are shown in Figs. 6C and 6D, respectively. The U-shaped pattern evident in the data in Figs. 6C and 6D indicate that asymmetries in receptive fields are more common at the lowest and highest (for the range of frequencies considered here) frequencies. Analysis of data in Figs. 6A and 6B, however, did not show statistically significant differences. Similar results were obtained from the analysis of data in Figs. 6C and 6D.

Scatter plots of standard deviations of first-spike latency (FSL) against CF for high-sync neurons and ANFs in cats are displayed in Fig. 6E. Low- and mid-spontaneous ANFs have the lowest standard deviations among ANFs (red circles in Fig. 6E). High-sync neurons, however, exhibit on average the lowest value in the variability of the FSL, as shown in Fig. 6E. Statistical analysis indicates significant differences significant (*p*<0.01, two-sample Kolmogorov-Smirnov test) in their values for the two data populations in Fig. 6E.

**Figure 7:**
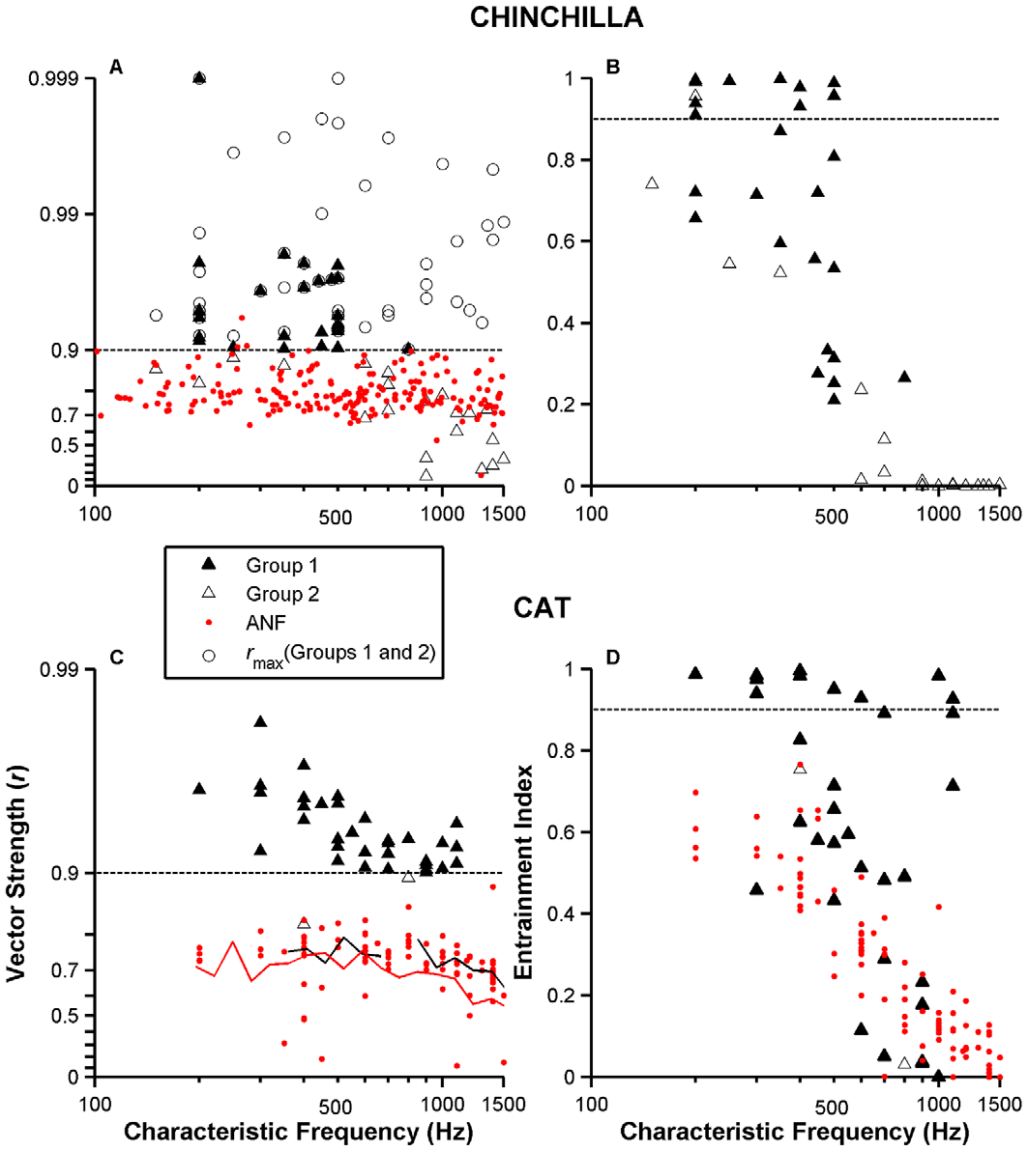
Enhancement of vector strength and entrainment in VCN neurons' discharges relative to ANFs'. Filled triangles in panels A and B denote vector strength values and entrainment indices, respectively, obtained from responses to 60-dB SPL tones at CF of a population (N = 26) of high-sync neurons (i.e., Group 1) in chinchillas. Similar results are in panels C and D for a population (N = 34) of cat high-sync neurons. Black and red continuous lines in panel C represent average vector strength values for ANFs in cats and chinchillas, respectively. Results of the same temporal analysis performed on Group 2 neurons are shown using open triangles in panels A–D (N = 21 in chinchillas; N = 2 in cats). Open circles in A represent *r*_max_values for high-sync and Group 2 neurons (see text). Red dots in panels A and C show *r*_max_ data for a population of chinchilla (N = 207) and cat (N = 63) ANFs. Red dots in panel D represent entrainment indices for ANFs.

Vector strength values computed from responses to CF tones are shown for a population of chinchilla and cat VCN neurons (Group 1: high-sync neurons) using filled triangles in Figs. 7A and 7C, respectively. Tone levels were usually 60-dB SPL. The CF range (up to ≈550 Hz) for chinchilla high-sync neurons is narrower than the range in cat VCN neurons, which includes neurons with CFs up to 1 kHz. Open triangles in Fig. 7A represent *r* values of Group 2 neurons (i.e., neurons with *r*>0.9 in their responses to single tones at certain off-CF frequencies, but not for stimulus frequencies equal to CF). Open circles in Fig. 7A display maximum *r* values as a function of CF (i.e., the stimulus frequency that elicits *r*_max_ is not necessarily equal to CF) for neurons in Groups 1 and 2. (Values indicated by the open circles in Fig. 7A represent *r*_max_ obtained from receptive field data.) Red dots in Fig. 7A represent *r*_max_ values for a population of chinchilla ANFs [9] and are analogous to the aforementioned open circles. That is, each red dot in Fig. 7A represents the largest vector strength measured from a family of responses of a neuron to single tones in the 100–1500 Hz frequency range. Therefore, the frequency of the tone that evoked *r*_max_ is not necessarily equal to CF. With the exception of three data points, *r*_max_ values measured from ANF responses are always <0.9. Red dots in Fig. 7C represent *r* values computed from responses to CF tones. Distributions of *r* values for ANFs and VCN neurons are almost non-overlapping and statistically significant (*p*<0.01, two-sample Kolmogorov-Smirnov test). Black and red continuous lines in Fig. 7C present average *r*_max_ values for cat and chinchilla ANFs, respectively.

Entrainment indices as a function of CF computed from responses to CF tones in chinchilla and cat VCN neurons are shown in Figs. 7B and 7D, respectively. VCN neurons in both species can evoke responses that are highly entrained (i.e., entrainment index >0.9). The main difference, however, lies in the CF range of neurons that can yield such responses. In the case of the cat, responses of neurons with CFs up to 1 kHz can show entrainment, which contrasts with the 500–600 Hz upper limit in the chinchilla. It is also evident that entrainment in VCN neurons is larger than in ANFs (dots in Fig. 7D).

### Distortion in the temporal representation provided by high-sync neurons

Although information about the stimulus period provided by entrained responses of high-sync neurons (e.g., Fig. 1) is highly accurate and with little variability, the representation of the stimulus waveform provided by neuronal discharges is usually distorted [19]. In fact, previous studies of the representation of the stimulus waveforms using spectral analysis of regular neuronal discharge has revealed a large amount of *distortion* [20], [21], [29] in such representation. This is because the entrained response to sinusoidal stimulation contains not only significant amplitude at the stimulus frequency but also at its harmonics—a consequence of the regularity of the time between consecutive neural discharges.

**Figure 8:**
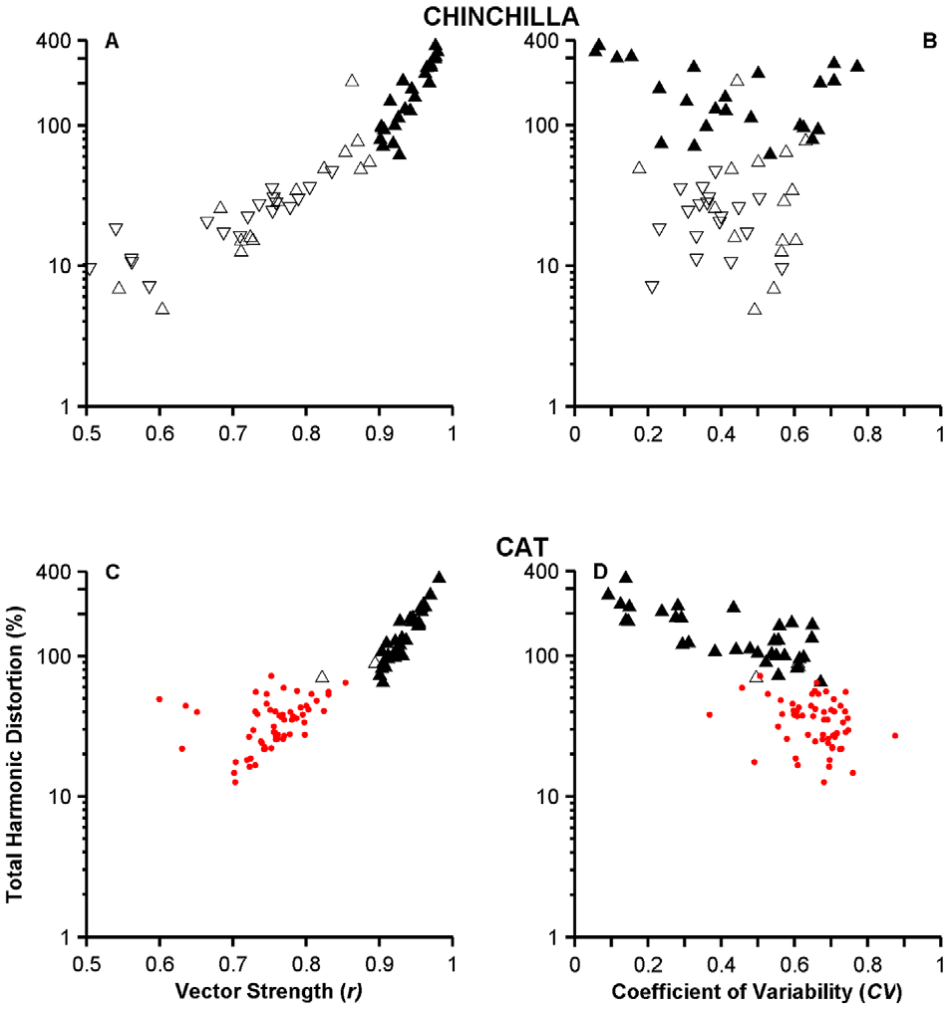
Distortion as a function of synchrony and interspike interval variability. Black filled (N = 26 and 34 in chinchillas and cats, respectively) and open “up” triangles (N = 16 and 2 in chinchillas and cats, respectively) represent data from high-sync and Group 2 VCN neurons, respectively. “Down” triangles represent data from Group 3 VCN neurons (N = 18). Red dots show cat ANF data (N = 59). Data from chinchillas are in panels A and B, and from cats in panels C and D.

Results in Fig. 1 indicate that high-sync neurons carry information about the stimulus waveform with higher distortion than ANFs. Period histograms obtained from responses of ANFs to single tones, such as those in Fig. 1, have a more sinusoidal shape than those computed from responses of high-sync neurons. Neurons with the highest THD values in Fig. 1 had the lowest *CV*s. Results in Fig. 1 indicate that high-sync neurons carry information about the stimulus waveform with higher distortion than ANFs. Neurons with the highest THD values in Fig. 1 had the lowest *CV*s. Results from the population analysis, however, show a more complex relationship between distortion and interspike variability (Figs. 8B and 8D), as measured using the *CV*. Although THD estimates computed from responses of chinchilla high-sync neurons (filled triangles in Fig. 8B) are higher than those obtained from VCN neurons in Groups 2 and 3 (open triangles in Fig. 8B), THD estimates are independent of their *CV* values, regardless of the type of VCN neuron. Similarly in cats, THDs for high-sync neurons are larger than those yielded by ANFs (Fig. 8D). *CV* values computed from the responses of some high-sync neurons, however, resemble those computed from the responses of ANFs.

Scatter plots of THD-vs.-*r* (Figs. 8A and 8C) show a near increasing monotonic relationship, at least for *r*>0.5. THD computations for lower synchronization values are less reliable due to very low signal-to-noise ratios. Results in Fig. 8 thus demonstrate that distortion in the neural representation by ANFs and VCN neurons is not related to interspike variability.

### Responses to vowel sounds

**Figure 9:**
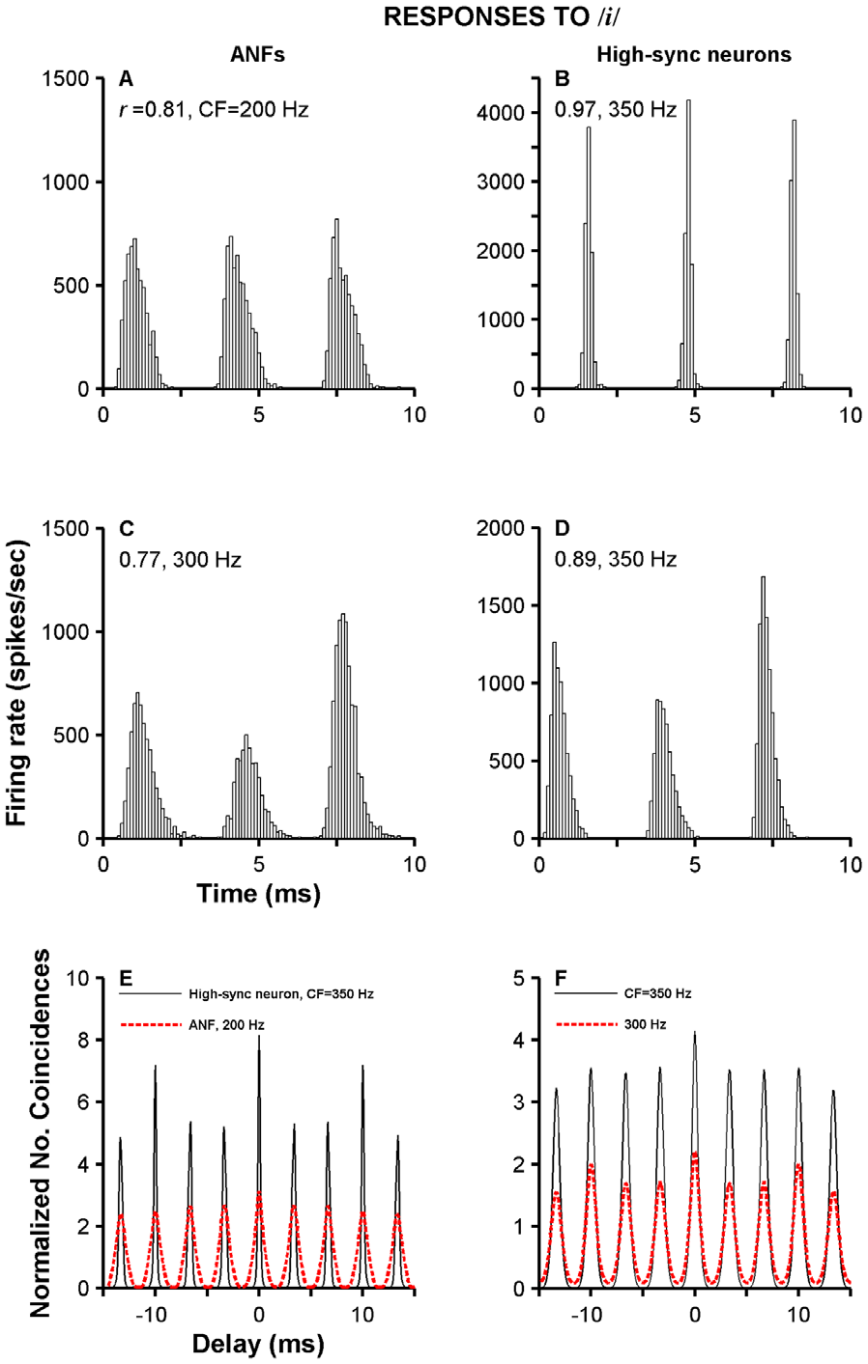
Responses to synthetic vowel sounds. Panels A and C exhibit period histograms obtained from ANF responses to the /*i*/ sound. Responses of two high-sync neurons to the same stimulus are shown in panels B and D. *r* values indicate strength of phase locking to *f*_0_ ( = 100 Hz). Panels E and F contrast SACs computed from high-sync neurons' (thin lines) and ANFs' (red thick lines) responses to /*i*/. Spike trains used to compute SACs in panel D are the ones used in panels A and B. Similarly, SACs in E and F were computed from response whose histograms appear in panels C and D.

Neural responses to synthetic and spoken whispered vowels were obtained in chinchillas and cats, respectively. Examples of a temporal analysis performed on two ANFs and two high-sync neurons are shown in Fig. 9. Period histograms (period = 1/*f_0_*) in Figs. 9A and 9C were computed from the responses to the vowel /*i*/ of two ANFs in chinchillas. Vector strength values in each plot represent the strength of phase locking to the frequency of F_1_ ( = 300 Hz). Individual peaks in period histograms of high-sync neurons' responses (Figs. 9B and 9D) are narrower than peaks in histograms of ANFs (Fig. 9A and 9D). *r* values computed from the responses of the two high-sync neurons are higher than the values computed from ANF responses. The two aforementioned properties of high-sync neurons are consequences of their outstanding temporal resolution.

Figures 9E and 9F exhibit normalized SACs obtained from ANFs' (red dashed lines) and high-sync neurons' responses (thin lines). (SACs in Fig. 9E were obtained from the same data used to compute the period histograms in Figs. 9A and 9B; similarly, Fig. 9F was computed from data used to construct histograms in Figs. 9C and 9D.) SACs depicted in Figs. 9E and 9F consist of a 300-Hz oscillation (i.e., three periods in 10 ms). In other words, there is a representation of the frequency of F_1_ in the responses of ANFs and high-sync neurons in Fig. 9. With the exception of the peak at the origin, the largest peak in the SACs occurs at a time lag corresponding to 10 ms, i.e., the period of *f*_0_. The largest peak in a SAC always occurs at the origin and its amplitude is referred to as the correlation index [7]. Uncorrelated spikes yield a unit value in a normalized SAC. Individual peaks in SACs computed from high-sync neurons' responses were narrower than those obtained from ANFs. Bandwidths of the central peaks, evaluated at half of their maximum value, for the high-sync SACs in Figs. 9E and 9F were 0.25 and 0.67 ms. Bandwidths of ANFs in Figs. 9E and 9F were 0.92 and 1.03 ms.

**Figure 10:**
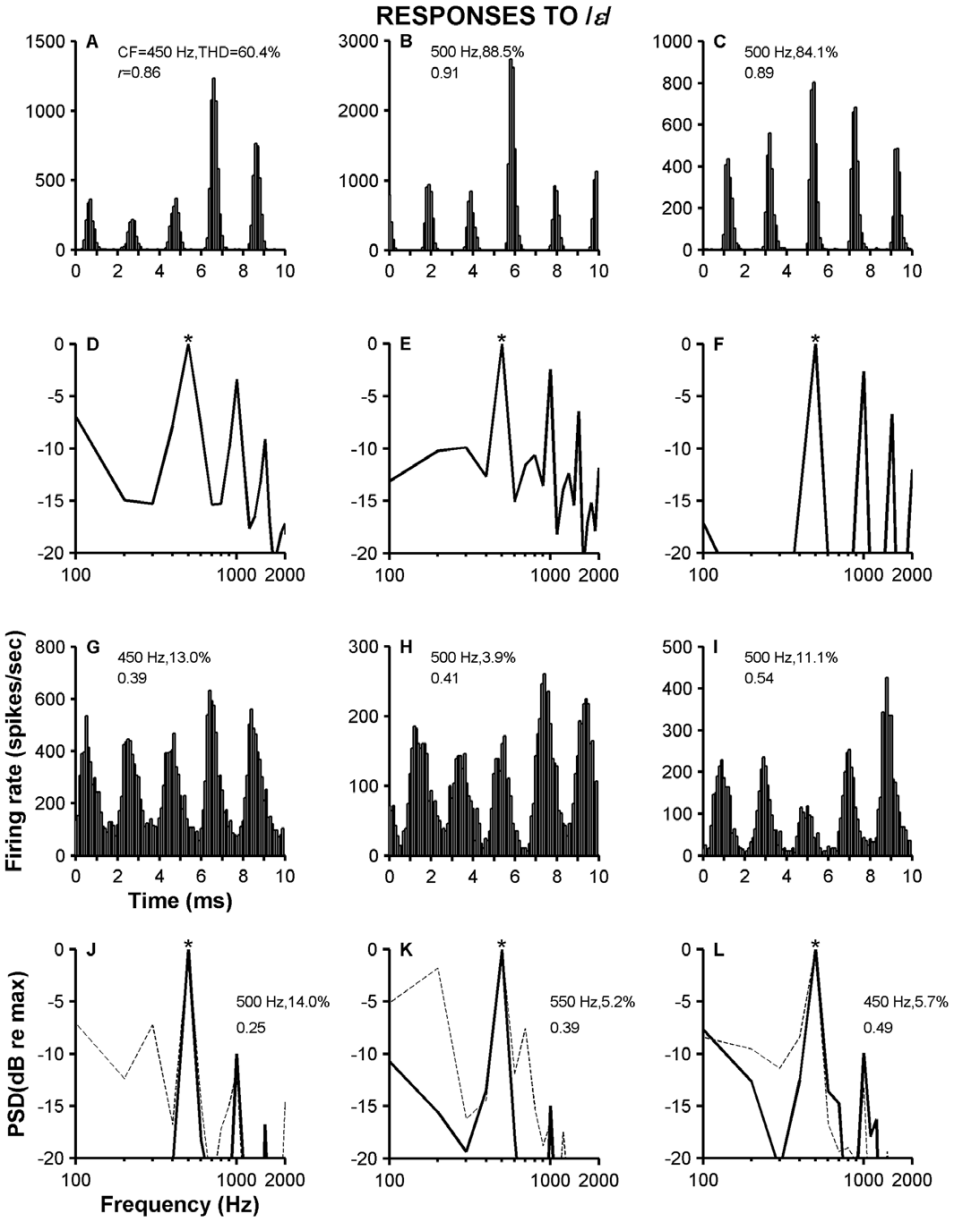
Temporal and spectral analyses of responses to /ε/ of high-sync and Group 3 VCN neurons. Panels A–C display period histograms obtained from responses of three high-sync neurons whose CF is indicated in each panel. Panel A recordings originate from the ventral acoustic stria. Panels G–I contain period histograms computed from responses of three VCN neurons (Group 3; CF indicated in each panel). Panels D–F (black continuous lines) and J–L (black continuous lines) show result of Fourier analysis obtained from responses shown in panels A–C and G–I, respectively. Dashed lines in panels J–L illustrate PSDs from three other VCN neurons (Group 3; CF indicated in each panel).

Figure 10 contrasts the neural representations of the vowel /*ε*/ in the responses of high-sync and Group 3 VCN neurons. Period histograms derived from responses to /*ε*/ of three high-sync neurons are shown in Figs. 10A–10C. The duration of each period histogram, 10 ms, equals the period of *f_0_*. The frequency of F_1_ ( = 500 Hz) is similar to the neurons' CF, which is listed on top of each figure along with THD and *r* values. (THDs were computed using the spectral amplitudes at F_1_, 2*F_1_, etc.). Each histogram consists of five peaks, which indicates a tendency of those neurons to follow the frequency of F_1_ ( = 5*f_0_). Fourier analysis of the period histograms in Figs. 10D–10F shows a predominant spectral component at the frequency of F_1_, which is marked with a star. There are also spectral peaks at frequencies corresponding to the harmonics of F_1_ (rectifier distortion products) [30], [31].

Period histograms obtained from responses of three VCN neurons (Group 3) to /*ε*/ are shown in Figs. 10G–10I and their respective Fourier transform amplitudes are shown in Figs. 10J–10L (thick continuous lines). Spectra from other neurons with similar CFs are also included in Figs. 10J–10L (thin dashed lines). All the responses—either in the time or frequency domains—exhibit a tendency of the neurons to follow the frequency of F_1_. Shapes of the peaks in the histograms of Group 3 neurons' responses appear broader and more “sinusoidal” than peaks in the response histograms of high-sync neurons. THD values in the responses of high-sync neurons were higher than the distortion carried in the responses of other types of VCN neurons. Likewise, *r* values computed at the frequency of F_1_ are also the largest for high-sync neurons.

**Figure 11:**
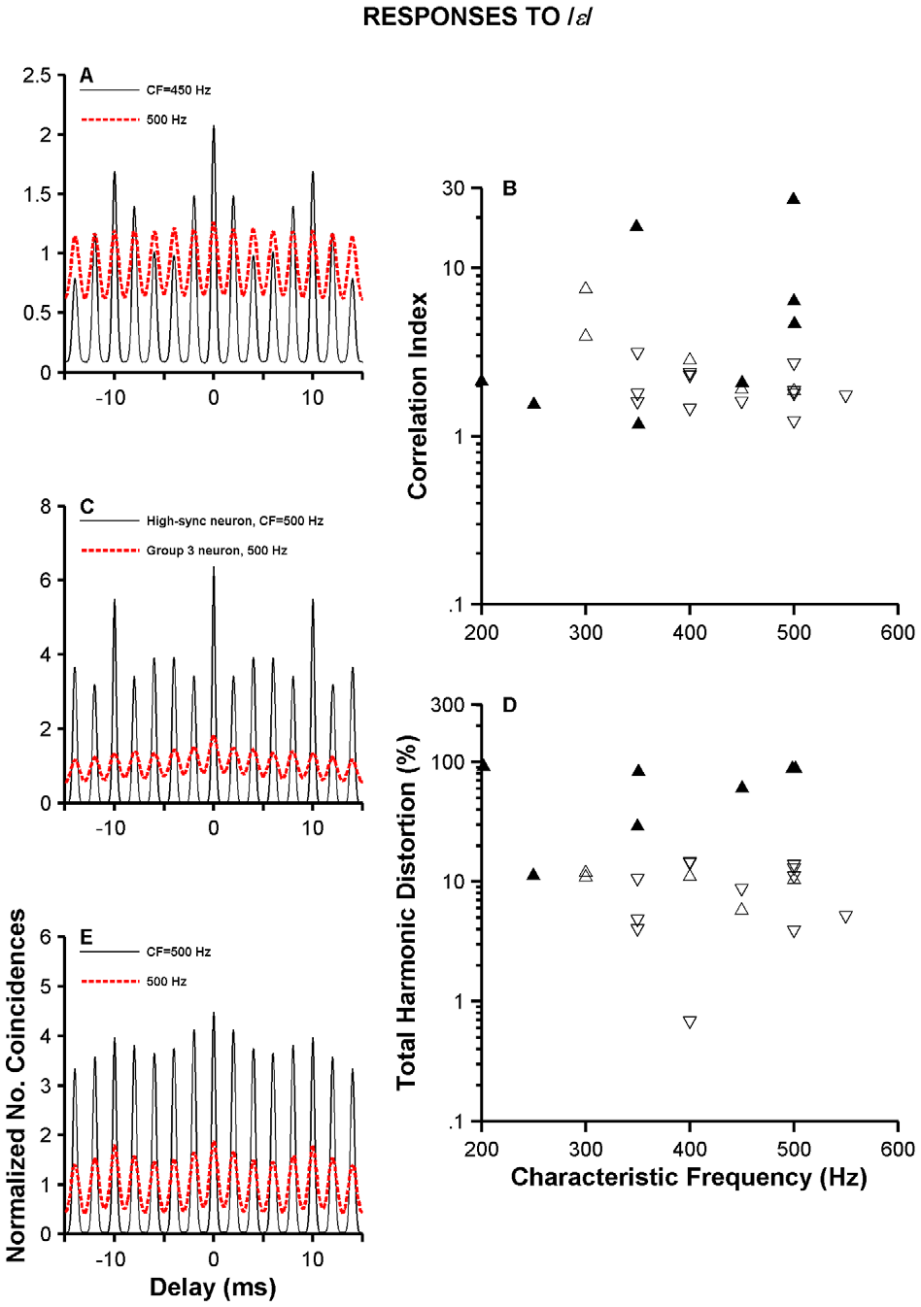
Normalized SACs and harmonic distortion of VCN neurons' responses to /ε/. Panels in A, C and E contain normalized SACs constructed from high-sync neurons (thin lines) and Group 3 neurons (red thick lines) responses to /ε/. High-sync neurons in A, C and E are the same neurons whose histograms are shown in Figs. 10A–10C, respectively. Group 3 neurons in panel A, C and E are the same as in Figs. 10–10C. Panel B displays a scatter plot of CIs as a function of CF for high-sync (filled triangles, N = 13), Group 2 (open triangles, N = 6) and Group 3 VCN neurons (inverted triangles, N = 13). Panel C displays THDs for the same neurons in panel B.

Figure 11 contains the results of the temporal and distortion analyses performed on VCN neurons' responses to /*ε*/. Thin lines in Figs. 11A, 11C and 11E represent SACs computed from the responses of neurons whose period histograms are in Figs. 10A, 10B and 10C, respectively. Normalized SACs constructed from the neurons' responses used in the histograms in Figs. 10G, 10H and 10I are plotted with red thick lines in Figs. 11A, 11C and 11E, respectively. SACs of high-sync neurons in Fig. 11 reveal the ability of those neurons to encode in their spike times the frequencies corresponding to *f*_0_ and F_1_, as indicated by the oscillations' periods and the larger size of the oscillations at 10 ms. By contrast, SACs of the Group 3 neurons consist mostly of a sinusoid with period equal to 1/F_1_. Individual peaks in SACs of high-sync neurons are also narrower than peaks in SACs of Group 3 neurons. CIs and THDs of high-sync and Group 2 neurons (“up” triangles in Figs. 11B and 11D, respectively) are larger in general than the corresponding values in Group 3 neurons (“down” triangles in Figs. 11B and 11D, respectively).

**Figure 12:**
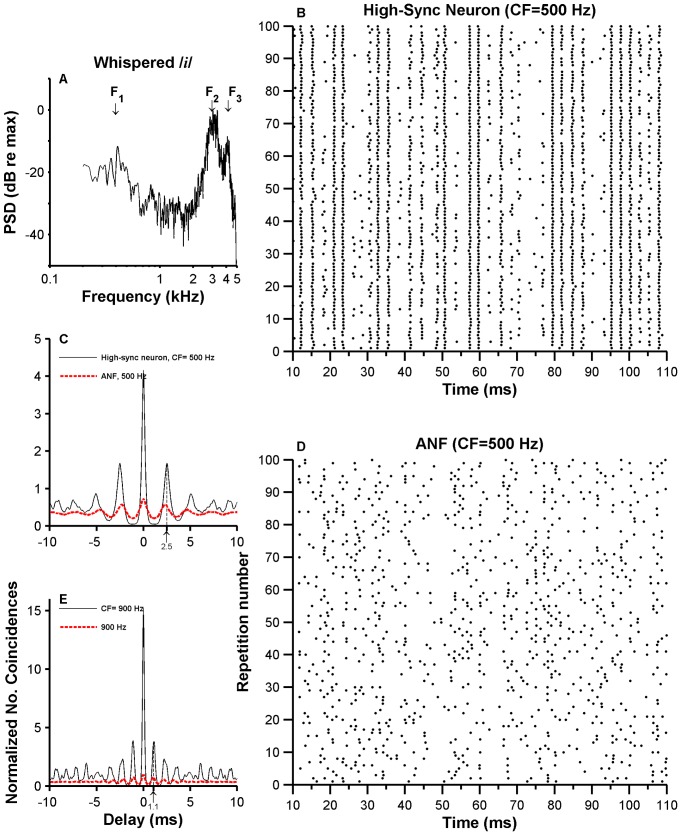
Responses of high-sync neurons and ANFs to the whispered /*i*/ sound. Panel A display the result of the PSD analysis of the whispered **/** *i* **/** vowel. F_1_, F_2_ and F_3_ represent the approximate location of the respective formants. Panels B and D show dot raster plots of the responses of a high-sync neuron and an ANF, respectively. SACs obtained from responses in B and C are shown in panel C (thin line: high-sync neuron; red thick line: ANF). SACs in panel E were obtained from data from another high-sync/ANF pair, with same CF ( = 900 Hz).

VCN neurons' and ANFs' responses to whispered vowels were registered only in the cat. Only responses to /*i*/ will be presented here since they constitute the most complete set of recordings. (Fig. 12A shows the PSD analysis for this vowel.) Dot raster plots of the responses of a high-sync neuron and an ANF to /*i*/ are shown in Figs. 12B and 12D, respectively. The dot raster plot of the high-sync neuron's responses consists of a series of vertical lines occurring at approximately 2.5 ms intervals (or multiples of this number), as easily appreciated in the three lines occurring between 95 and 100 ms (Fig. 12B). This contrasts with the apparently random nature of the ANF responses (Fig. 12D). SACs computed from the responses of those units are shown in Fig. 12C. Both SACs in Fig. 12C consist of a damped oscillation whose periodicities are approximately equal to the inverse of each of the fiber's CF. Individual peaks in the high-sync neuron's SAC (thin line in Fig. 12C) are narrower and have higher values than peaks in the ANF's SAC (red thick line in Fig. 12C). SACs constructed from the responses to /*i*/ of two other fibers with higher CFs (around 900 Hz) are shown in Fig. 12E. Although the overall shape of both SACs resembles those in Fig. 12C, the oscillation's periodicity varies in proportion to the fiber's CF. Temporal resolution in SACs of high-sync neurons is higher, as indicated by the narrower and higher individual peaks, than in SACs of ANFs. A similar enhancement in temporal features was also obtained from the responses to broadband noise of high-sync neurons and ANFs [7]. The enhancement in temporal processing was quantified using the “higher peak height or CI” [7].

**Figure 13:**
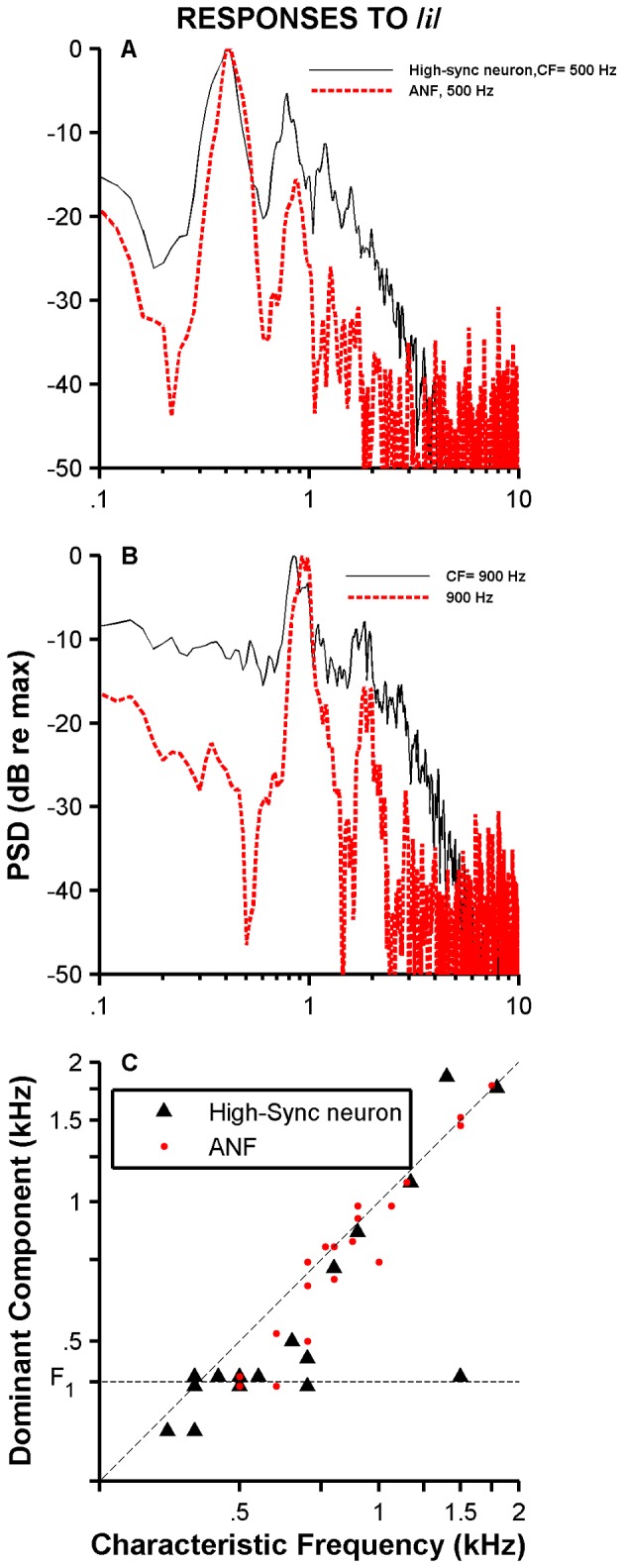
PSD and dominant-component analyses of SACs. Panels A and B show Fourier transform amplitude of SACs displayed on Figs. 12C and 12 E, respectively, using the same color convention. Panel C exhibits a scatter plot of the frequencies of dominant components as a function of CF for a population of high-sync neurons (N = 19) and ANFs (N = 24).

Figures 13A and 13B show the Fourier transform amplitudes of the shuffled autocorrelograms (i.e., PSDs) shown in Figs. 12C and 12E, respectively, using the same conventions of color and line thickness. Maximum values of the two PSDs shown in Fig. 13A happen at frequencies near the frequency of F_1_. PSDs displayed in Fig. 13B, however, have maximum values occurring at approximately 900 Hz—near the units' CFs. Amplitudes of the largest spectral peaks relative to the second largest ones are lower for the responses of high-sync neurons than for ANFs (Figs. 13A and 13B). This indicates a larger distortion in the representation provided by high-sync neurons than the one provided by ANFs. Figure 13C contains a scatter plot of the frequencies of the maximum values of the PSDs—i.e., the “dominant components” [32]—as a function of CF. For units with CF<700–800 Hz, dominant components in the responses of ANFs and high-sync neurons are “locked” to the F_1_ frequency. Dominant components in the response of neurons with CFs>800 Hz, however, are similar to the neuron's CF.

**Figure 14:**
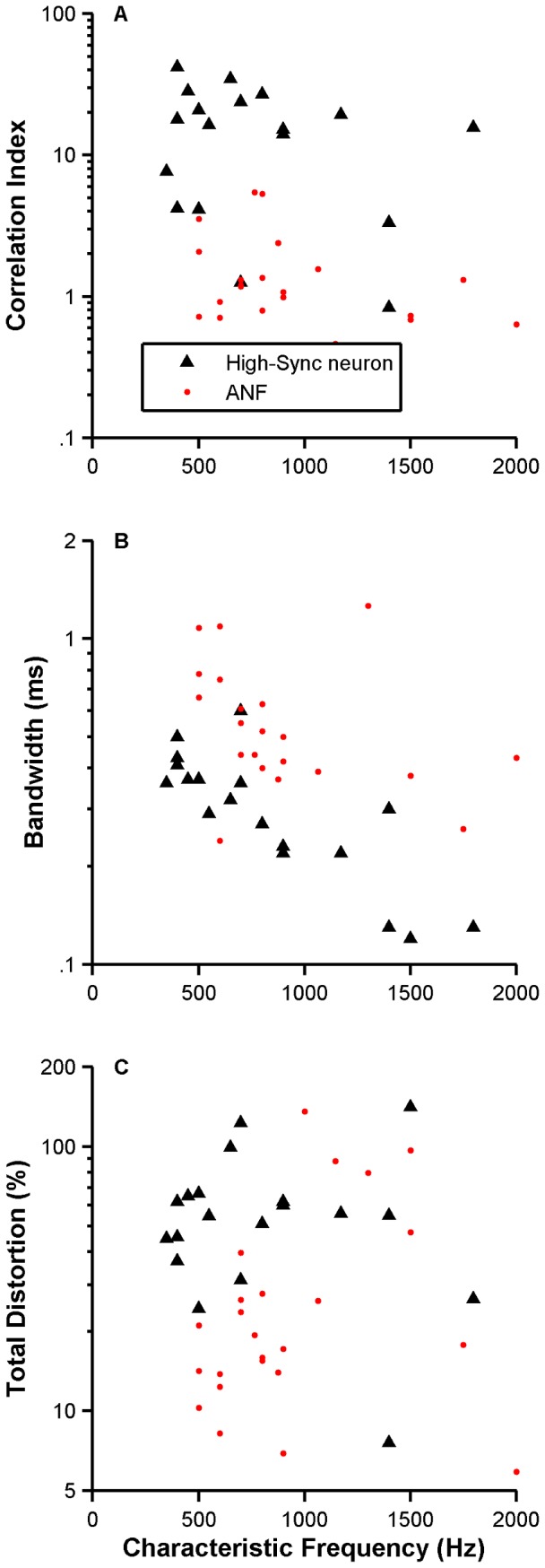
Temporal processing of the whispered /*i*/ vowel by high-sync neurons and ANFs. Panels A and B, respectively, display amplitudes and bandwidths of the central peaks in SACs computed from responses of cat high-sync neurons (black triangles, N = 19) and ANFs (red dots, N = 24) to the whispered /*i*/ sound. The results of the distortion analysis performed on the responses of high-sync neurons and ANFs are shown in panel C.

Figure 14A displays CIs measured from responses to the whispered /*i*/ vowel of a population of high-sync neurons (filled triangles) and ANFs (red circles). CI estimates obtained from high-sync neuron responses are usually larger than those computed from ANF responses. Conversely, bandwidths of the central peak in the SACs of high-sync neurons are generally lower than those originating from ANFs' responses (Fig. 14B). The distortion in the representation of the vowel /*i*/ is also larger for high-sync neurons than for ANFs (Fig. 14C).

## Discussion

This report documents for the first time the existence of highly synchronized neurons in the VCN of the chinchilla as well as the responses of high-sync neurons to vowel sounds in chinchillas and cats. The use of high-impedance glass pipettes in this research to record extracellular action potentials from the VCN introduced difficulties in distinguishing between responses of ANFs and VCN neurons, but at the same time made it possible to record from high-sync neurons without electrical contamination [6]. The use of metal electrodes would avoid ANF recordings [33], but that type of electrode can pick up unwanted local-field potentials when recording from units with high amounts of phase locking.

Responses of highly synchronized neurons to CF tones exhibit vector strength values above 0.9, which represents an improvement over the synchronization limits of ANFs (Fig. 7). Preliminary results in the encoding of synthetic vowels in the temporal structure of the responses of high-sync neurons also indicate an enhancement relative to the AN's temporal processing (Fig. 9).

An enhancement of synchrony was also evident in the responses to tones and whispered vowels of high-sync neurons in the cat. Responses of high-sync neurons and ANFs to the whispered /*i*/ value show differences in the temporal structures of their responses. Shuffled autocorrelograms constructed from the responses of high-sync neurons exhibit a better temporal precision than SACs of ANFs. FSLs computed in the responses to tones in cat high-sync neurons are smaller than FSLs in the responses of ANFs.

### Temporal properties of highly synchronized neurons in chinchilla and cat

Responses to CF tones of high-sync VCN neurons in the chinchilla consist of trains of action potentials that are well synchronized to one phase of the stimulating waveform (Figs. 1 and 2). For CFs<500–600 Hz, *r* values computed from responses to CF tones can be above 0.9 (Fig. 7A). By contrast, ANF responses to CF tones in that frequency range are always below 0.9 (Fig. 7A, [9],[10]). These results indicate that highly synchronized neurons do exist in the chinchilla and provide an enhancement in the temporal representation provided by ANFs. (Cat ANF responses to single tones can also be highly synchronized to the stimulus frequency [i.e., *r*>0.9] provided that the stimulus frequency is well below the unit's CF [24], [28].)

Plots of vector strength as a function of CF for highly synchronized neurons in the chinchilla display a low-pass characteristic with a cutoff frequency of around 500–600 Hz (Fig. 7A). This is lower than in the cat (Fig. 7C), where CFs of high-sync neurons can be as high as 1000 Hz. Responses of ANFs in cats and chinchillas evoke similar *r*_max_ values for CF<600 Hz (inset in Fig. 7A [9]). For CFs>600 Hz, however, *r* values in cats are larger than in chinchillas. Therefore, the reduced CF range of high-sync neurons in chinchillas could be a consequence of temporal processing limitations in the auditory nerve of the chinchilla.

Some high-sync VCN neurons fire one action potential at almost every stimulus period (Figs. 1B, 3A and 4A). In other words, the responses of those neurons are entrained to the stimulus frequency. Entrainment has been shown previously by Joris et al. [6] and Rhode [24] in the responses of cat VCN neurons, but never before in a rodent. Entrainment to CF tones occurs in the chinchilla up to CF<600 Hz (Fig. 7B).

Standard deviation of FSLs measured from responses to CF tones of high-sync neurons are below 1–2 ms (Fig. 6E) and also below the σ_FSL_ measured in most ANF responses. This decrease in the variability of the time of occurrence of the first spike in response to a tone represents another type of temporal enhancement of ANF responses.

Responses to CF tones of high-sync neurons in the cat yield *r_max_* values that are larger than those measured in ANFs (Fig. 7B). This enhancement has been found by some authors [6], [7], [17] but not others [11], [13]–[16]. Considering the large amount of data collected by those authors using the same laboratory species, it is difficult to explain the differences in their findings. It is possible, however, that previous studies [11], [13]–[16] did not target the VCN region(s) in which high-sync neurons seem to be located. Spherical and globular bushy cells, which are capable of high synchronization and entrainment [6], [19], [24], are found mostly in the nerve root area and in the rostral end of the AVCN, respectively [34]. In addition, because of phase-locked local field potentials, the common use of metal electrodes to record from the VCN might have prevented the recordings of high-sync neurons in previous studies. Joris and colleagues [6], [7], [19] recorded from the ventral acoustic stria, one of the three CN output tracts, which allowed for the sampling of AVCN neurons, especially from bushy cells.

The proportion of chinchilla high-sync neurons (26%, 26/99) is much lower than the percentage (75%) found in cats [6] for the same CF range. One possible explanation for this discrepancy is that whereas recordings performed by Joris [6] originated mostly from bushy cells, in the present study no particular VCN region was targeted and thus fewer bushy cells were sampled.

### Discharge rate properties of highly synchronized neurons

High levels of neural synchrony and entrainment in high-sync neurons affect their firing-rate profiles. Receptive fields in Figs. 2E, 2F, 3E, 3F, 4E and 4F contain maximum firing rates similar to the stimulating frequency. (For example, the maximum firing rate in Fig. 3F is approximately 350 spikes/s, which corresponds to the stimulus frequency value.) Depending on the neuron's CF, the frequency that evokes such high firing rates could be either below or above CF.

Receptive fields of neurons with a certain level of spontaneous activity might exhibit firing rates that are below the spontaneous activity of the neuron. One consequence of the firing rate reduction is that receptive fields of high-sync units are narrower than those of ANFs, especially at medium to high intensity levels. This decrease in firing rate in response to a single tone is probably due to inhibitory mechanisms occurring at off-CF frequencies. It is likely that inhibitory process also occur at CF frequencies, at least in chinchilla, since approximately 30% of units classified as Group 1 or 2 in chinchillas have RIFs that were considered nonmonotonic.

Shifts in the center of gravity of receptive fields in ANFs of the chinchilla have been shown before [9]. Changes in firing rate (ΔR_max_) are much larger in the VCN than in the auditory nerve (Fig. 1). Such differences in firing rate probably stem from entrainment properties of high-sync neurons as well as inhibitory mechanisms described in the previous paragraph. Profiles of shifts in the center of gravity as a function of CF shown in receptive fields of ANFs indicate a zero shift at around 1 kHz [9], which is different from the equivalent value for VCN neurons (around 500–600 Hz in Fig. 6A).

### Highly synchronized neurons in other rodent species

Previous studies of temporal processing by AVCN neurons in the guinea pig [16] and rat [18] indicate that vector strength values computed from responses to CF tones are <0.9. Vector strength values (i.e., mean plus one standard deviation) computed from responses of spherical bushy cells in gerbil (Fig. 5 in [35]) are lower than those shown here for high-sync neurons (Figs. 7A and 7C). An apparent small percentage of individual *r* values in another recent study in the gerbil (Fig. 8 in [36]) are above 0.9. It is not clear from the two aforementioned studies, however, what the *r* values are in responses to CF tones. Nevertheless, works by Dehmel et al. [35], Kuenzel et al. [36] and Paolini et al. [18] demonstrate an enhancement in temporal processing by VCN cells relative to ANFs, in spite of their apparent lower vector strength values.

Nonmonotonic RIFs have been found in AVCN units in the gerbil [37], presumably due to the overlapping of postsynaptic inhibitory areas with excitatory ones. The proportion of nonmonotonic RIFs in chinchillas is smaller than (30%) but comparable to that found in gerbils (almost 50%). Results from another gerbil study [38] showed only monotonic RIFs, probably because recordings in [38] were performed in the caudal end of the AVCN and those in [37] originated from the rostral end of the AVCN.

All the RIFs measured in high-sync units in cats were monotonic, yet inhibitory sidebands were present in some units (e.g., Fig. 4E).

### Morphology of highly synchronized neurons

Labeling experiments [6], [24] have indicated that globular and spherical bushy cells show high neural synchronization and entrainment values. PSTHs and receptive fields of high-sync neurons shown in this work resemble low-CF neurons described by Rhode and Smith [11]. Those neurons, which were named “onset units with low CFs” (O_lf_) by Rhode and Smith, have high *r* values (in the 0.8–0.99 range) and can entrain to stimulus frequencies up to around 1 kHz. The authors thought of those neurons as representing “the low end of the frequency spectrum of onset cells.” Octopus cells in cats, whose responses to sound display an onset pattern, are located in the posterior end of the ventral cochlear nucleus [11], [39]. It appears that low-CF onset units and high-sync neurons, which are thought to be bushy cells, share many characteristics in their responses to tones and broadband stimulation.

Although marking of the recording site was not done in this study, placement of the electrode was documented, and based on histological processing in some animals it was judged that recordings originated from either the posterior end of the AVCN or the nerve root area. Because globular bushy cells in the chinchilla are located in the anterior end of the PVCN and the posterior end of the AVCN [40], VCN recordings in this work likely originate from globular bushy cells. Recordings from the rostral end of the AVCN have yielded units with strong phase-locking in their responses to CF tones, but their vector strength values were not published [41].

### Possible mechanisms of synchronization enhancement

Results of computer simulations have shown that a “coincidence detection” mechanism is capable of enhancing synchrony and entrainment [19]. A postsynaptic neuron implementing this type of mechanism generates an action potential if a certain number of inputs arrive at the same time or within a given time window of very short duration. A relatively large number of inputs is needed for coincidence detection [19], which makes it viable as a model for globular bushy cells. Such neurons receive a large amount of ANF inputs.

Spherical bushy cells, which receive inputs from only a few auditory nerve fibers, also show enhanced synchronization over ANFs, at least in cats [19]. Simulating the synchronization enhancement properties of spherical bushy cells using coincidence detection has been proven difficult [19].

### Distortion in the representation of the stimulus waveform by high-sync neurons

The representation of the stimulus waveform by a train of action potentials is usually distorted [21]. There are many ways to provide a quantitative measure of the distortion in the representation of the stimulus waveform. When the stimulating waveform is periodic, for example, the distortion can be visualized using period histograms, such as those that appear in Fig. 1. The process of fitting a sinusoidal function to a given period histogram and then estimating the error in the fit would yield a measure of the distortion. In this manuscript Eq. 1 was used to quantify harmonic distortion. Figures 8,10, 11 and 14 display THD values for ANFs and VCN neurons. Although all units encode the stimulating waveform with relatively large amounts of distortion, high-sync neurons exhibit the largest amounts, thus agreeing with a previously published assertion [19].

Previous studies of the transmission of information by spike train carriers have linked interspike variability to high THD [20], [21]. For example, in response to a sinusoidal excitation, it has been argued [21] that Poisson processes carry information about the stimulus waveform with less harmonic distortion than “regular” point process, i.e., process with less variable interspike interval times.

Given that ANF discharges can be modeled reasonably well using modified Poisson processes [42], it was not surprising that THD values obtained from responses of high-sync neurons were larger than those estimated from ANF responses or other types of VCN neurons (Fig. 8). The relationship between THD and interspike variability, however, is a complex one. *CV*s of Group 2 neurons overlap those obtained from responses of high-sync neurons (Fig. 8B), yet THD values for the latter type of neurons are larger than for the former. There is also a considerable amount of overlap between the *CV*s obtained from the responses of ANFs and high-sync neurons in the cat (Fig. 8D), yet THDs measured in high-sync neurons are always the largest.

In general, distortion in the representation of the stimulus waveform in the neural responses presented in this work depends on the amount of synchronization to the stimulus frequency (Figs. 8A and 8C), rather than on interspike variability. As with the entrainment index and vector strength [6], THD and vector strength are different measures of the timing of the response. (THD computations are based on the results of a Fourier transformation of an autocorrelation function. Two neural point processes may have the same vector strength but different autocorrelation functions.)

### Responses to vowel stimuli

Synchronization to either *f_0_* or F_1_ is higher, on average, for high-sync neurons than for ANFs or other types of VCN neurons (Figs. 9 and 10). Temporal enhancement by high-sync neurons is thus also shown in their responses to speech-like stimuli. Period histograms based on responses of high-sync neurons are narrower than the peaks in the period histograms obtained from responses of other types of VCN neurons. This translates into a poorer representation of the stimulus waveform.

Responses of high-sync neurons and ANFs to the whispered /*i*/ vowel were measured in the cat. Although it has been suggested [43] that perceived pitch of whispered vowels is determined by one of the first two spectral prominences (formants F_1_ or F_2_), whispered vowels do not provide a compelling pitch percept because there is no *f_0_* (Fig. 12A). Temporal analysis of the neuronal responses was thus performed by estimating SACs, whose waveforms consist of a damping oscillation (Figs. 12C and 12E). The main periodicity in the oscillation was approximately the same as 1/CF (arrows in Figs. 12C and 12E). Dominant component analysis [32] performed on whispered-vowel responses of ANFs and high-sync neurons shows that the largest spectral component in the response occurs at the unit's CF (Fig. 13C). Certain features of SACs, such as CI and bandwidths of the central peak (Fig. 14), indicate that temporal processing in the responses to vowels by high-sync neurons is enhanced relative to the processing performed by ANFs.

### Functional relevance

Although it is generally thought that the speech envelope is the most important carrier of information, the temporal fine structure (TFS) of speech has been the subject of recent work (e.g., [44]) and might be particularly important for speech recognition in the presence of fluctuating background noise. TFS is encoded in the temporal structure of ANF responses and depends on the amount of phase locking to the stimulus waveform. Because high-sync units enhance the amount of phase locking present in ANFs, the role of high-sync units in encoding TFS might be considerable. In fact, results in Figs. 9–12 show that, in general, synchronization to F_1_ of vowels is higher for high-sync neurons than for ANFs. The regular timing in the response of high-sync neurons is evident in the spectral representation of the spike train. For example, amplitude of the spectral component at 2*F_1_ relative to the amplitude at F_1_ is larger for high-sync neurons than for ANFs.

The distorted representation of the stimulus waveform that is provided by high-sync neurons raises the question of the relevance of this type of neuron in the processing of environmental sounds—speech included. Globular and spherical bushy cells, whose responses to tones can be considered as those of high-sync neurons [19], [24], send their main projections to binaural brainstem nuclei involved in sound localization [45]. There is, however, anatomical and physiological evidence that suggest other roles for high-sync neurons. Globular bushy cells provide an excitatory input to the medial nucleus of the trapezoid body, which in turn projects to the ventral nucleus of the lateral lemniscus (VNLL), a predominantly monaural center involved in tasks related to temporal processing and pattern recognition of sounds [46], [47]. The low variability in FSLs measured in responses to tones of high-sync neurons might provide an indication of the ability of those neurons to detect stimulus changes.
